# Prenatal alcohol exposure is associated with altered feto-placental blood flow and sex-specific placental changes

**DOI:** 10.1172/jci.insight.186096

**Published:** 2025-02-10

**Authors:** Sarah E. Steane, Christopher Edwards, Erika Cavanagh, Chelsea Vanderpeet, Jade M. Kubler, Lisa K. Akison, James S.M. Cuffe, Linda A. Gallo, Karen M. Moritz, Vicki L. Clifton

**Affiliations:** 1 The University of Queensland, St Lucia, Queensland, Australia.; 2 Mater Research Institute, Woolloongabba, Queensland, Australia.; 3 Queensland University of Technology, Brisbane, Queensland, Australia.; 4 University of the Sunshine Coast, Petrie, Queensland, Australia.

**Keywords:** Development, Reproductive biology, Growth factors, Molecular biology, Obstetrics/gynecology

## Abstract

**BACKGROUND:**

Prenatal alcohol exposure (PAE) around conception in preclinical models results in placental insufficiency, likely contributing to offspring abnormalities. Altered placental DNA methylation (DNAm) and gene expression suggest epigenetic mechanisms, perhaps involving impacts on methyl donor levels. PAE around conception in women is common but placental effects are rarely examined. This cohort study investigated associations between PAE around conception and intake/plasma measures of the methyl donors folate and choline, feto-placental blood flow, and placental growth measures, gene expression, and DNAm.

**METHODS:**

Pregnant participants delivered at Mater Mothers’ Hospital, Brisbane, Queensland, Australia (*n* = 411). Dietary intake of choline and folate were calculated and plasma concentrations measured using mass spectrometry (MS) and clinical immunoanalyzer, respectively. Cerebroplacental ratio (CPR) was calculated using Doppler measurements. Placentas were weighed/measured at delivery and samples used to quantify methyl donors (MS), global DNAm (ELISA), and gene expression (quantitative PCR). Data were compared between control/abstinent and PAE groups, by fetal sex.

**RESULTS:**

A CPR <5th-centile, indicating fetal brain sparing because of placental insufficiency, was found in 2% of controls and 18% of the PAE group, mostly male fetuses (63%). Compared with controls, male PAE placentas had reduced mean thickness and placental growth factor mRNA and DNAm, whereas female PAE placentas had increased *S*-adenosylmethionine and a trend toward increased DNAm.

**CONCLUSION:**

PAE around conception is associated with reduced CPR and altered placental growth measures, particularly in males, with potential implications for future health.

**FUNDING:**

National Health and Medical Research Council (APP1191217) and Mary McConnel Career Boost Program for Women in Paediatric Research (WIS132020).

## Abstract 2

Alcohol consumption around conception can impact placental growth and function, compromising blood flow and potentially nutrient and oxygen supply to the fetus, particularly in males.

## Introduction

Prenatal alcohol exposure (PAE) increases the risk of early miscarriage (1), preterm birth, low birth weight (2), and stillbirth (3). PAE can also result in lifelong neurological and physical abnormalities known as fetal alcohol spectrum disorder (FASD), which is the most common preventable neurodevelopmental disorder in the United States (4). Up to 80% of pregnant women in some Western countries report alcohol consumption, often at binge levels, most commonly prior to pregnancy recognition, after which time most women cease (5, 6). Although the negative effects of PAE throughout gestation on neurodevelopmental outcomes are well documented (7), effects of PAE occurring only around conception have not been widely studied.

We have previously investigated effects of PAE around conception using a unique rat model of periconceptional ethanol exposure up until blastocyst implantation. This exposure resulted in fetal growth restriction (FGR) and metabolic and neurodevelopmental abnormalities in adult offspring, many of which occurred in a sex-specific manner (8–11). We also found sex-specific effects of PAE in the placenta at late gestation, including altered morphology, DNA methylation (DNAm), and gene expression (10, 12). Further studies in the same model found increased DNAm in preimplantation blastocysts, impaired trophoblast invasion into maternal spiral arteries, altered placental expression of growth factors, and reduced placental vascularization at midgestation (13). Other rodent models of PAE around the time of implantation have reported similar effects (14, 15). These studies suggest that PAE in early pregnancy produces a placental insufficiency phenotype, which may originate from altered epigenetic regulation of gene expression in the embryo. To date, these outcomes have not been investigated in a clinical population.

However, our recently published systematic review and meta-analysis across 33 clinical studies found associations between PAE and an increased likelihood of placental abruption and reduced placenta weight (16). Included studies examining placental morphology and gene expression found that PAE was associated with reduced expression of angiogenic proteins, including placental growth factor (PlGF) and vascular endothelial growth factor (VEGF) receptors (17). PAE was also associated with a reduced number of placental villi, reduced density of blood vessels within the villi, and reduced diameter of vessel lumens (17, 18). Whether such alterations affect placental blood flow has not been investigated in women. However, first-trimester PAE in nonhuman primates results in increased placental resistance to blood flow, measured using Doppler ultrasound (19, 20). An important limitation highlighted by our review was that placental sex was rarely considered in analyses. Only 1 study examined placentas separately by sex and found that PAE was associated with increased DNAm in male placentas, a finding that was not detected when male and female placentas were pooled (21). Sex-specific placental effects of PAE are important to understand, as they may contribute to the sex differences in outcomes identified among individuals with PAE (22). Another limitation identified by our review was that timing of PAE was often poorly defined; therefore, whether placental alterations originated from PAE in early pregnancy could not be determined. Nevertheless, the effects of PAE on placentas from women are consistent with the preclinical studies of PAE around conception only, described above (10, 12–15), suggesting that PAE may affect fetal development, at least in part, via placental insufficiency, involving altered epigenetic regulation of gene expression. However, this does not discount that PAE occurring later in gestation can affect the placenta, with implications for fetal development (23).

The mechanism through which PAE disrupts DNAm is not completely understood but has been hypothesized to involve effects on methyl donor availability (24). Folate and choline are dietary micronutrients that provide methyl groups for generation of the universal methyl donor *S*-adenosylmethionine (SAM) via the 1-carbon metabolism (1CM) pathway. Alcohol consumption reduces folate absorption and alters its metabolism, resulting in lower levels of SAM (25, 26). PAE has also been found to alter activity of the mechanistic target of rapamycin (mTOR) (27), which coordinates cell growth in response to nutrient availability, including regulation of folate transporter expression and synthesis of SAM (28, 29). However, choline can provide methyl groups for 1CM in the liver when folate is limited (30). During pregnancy, deficiency in either folate or choline has been found to exacerbate effects of PAE on placental and fetal outcomes, whereas supplementation can ameliorate some of these effects (reviewed in ref. 31). The impact of PAE on placental and fetal development may therefore be moderated by maternal intake of folate and choline.

**Figure 1:**
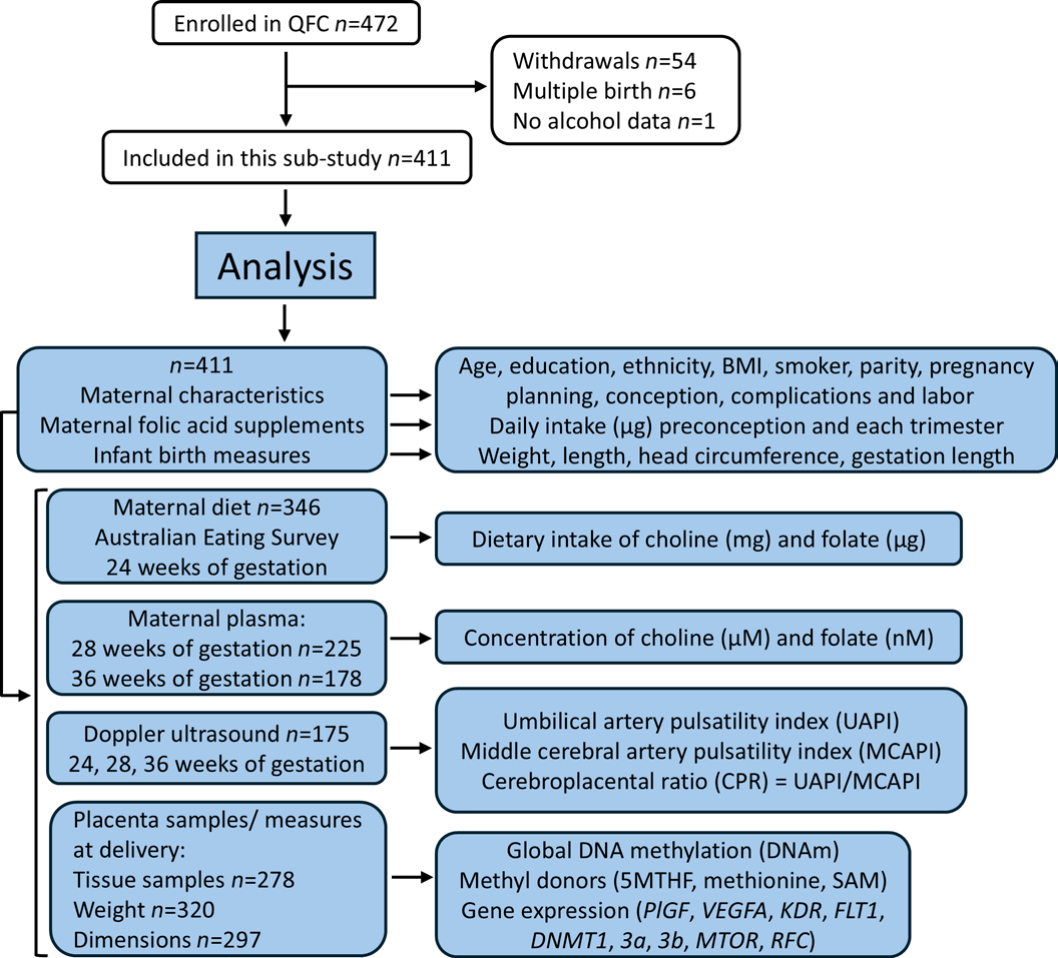
Flow diagram of study. Number of participants enrolled, withdrawals/exclusions, and final number included. Sample sizes for participant data/samples are shown with details of analyses. 5MTHF, 5-methyltetrahydrofolate; SAM, *S*-adenosylmethionine; *PlGF*, placental growth factor; *VEGFA*, vascular endothelial growth factor; *FLT1*, VEGF receptor 1; *KDR*, VEGF receptor 2; *DNMT*, DNA methyltransferase; *MTOR*, mechanistic target of rapamycin; *RFC*, reduced folate carrier.

The current study used data and samples collected from the Queensland Family Cohort (QFC) (*n* = 411, Figure 1), a prospective longitudinal birth cohort in Australia, to investigate associations between PAE and 1) maternal intake and plasma measurements of folate and choline; 2) Doppler measurements of feto-placental blood flow (cerebroplacental ratio; CPR); 3) infant and placenta growth measures; 4) placental mRNA expression of *PlGF*, *VEGF*, and *VEGFR*; 5) placental global DNAm, levels of methyl donors, and mRNA expression of DNA methyltransferases (*DNMT*s), *MTOR*, and reduced folate carrier (*RFC*). Fetal/placental outcome data were compared between the control/abstinent group and the PAE group overall and when divided by timing of exposure (preconception only, PC; or continuing during pregnancy, PCP). Based on findings of preclinical studies, data from male and female fetuses/placentas were analyzed separately.

## Results

### Maternal characteristics and outcome data

This study included QFC participants with singleton pregnancies and information on alcohol consumption (Figure 1). Mean age of participants was 32 years, and mean BMI was in the healthy range (Table 1). Few participants smoked (6.4%) or used recreational drugs (3.4%), and most (>70%) self-reported Caucasian race, were educated to tertiary level, and had a planned, uncomplicated pregnancy.

#### PAE.

PAE was reported by 73.5% of the cohort (Table 1), with 42.4% reporting exposure only in the 12 weeks preconception (PC group) and 31.1% reporting exposure preconception and during pregnancy (PCP group). Of the 128 women in the PCP group, 99 reported alcohol consumption preconception and trimester 1 (T1), and most of these women (*n* = 65) reported no further consumption beyond T1 (Supplemental Table 1; supplemental material available online with this article; https://doi.org/10.1172/jci.insight.186096DS1). Comparison of maternal characteristics between groups (Table 1) revealed that the PAE group had a higher proportion of Caucasian women (*P* < 0.001) and women with spontaneous labor (versus induced/cesarean delivery, *P* = 0.03) compared with the control group. There was a trend toward a lower proportion of women in the PAE group with pregnancy complications (*P* = 0.05) and with a parity of 2+ (*P* = 0.06) and a trend toward a higher proportion with tertiary education (*P* = 0.07). When the PAE group was divided by timing of exposure, both PC and PCP groups had a higher proportion of Caucasian women compared with controls (*P* < 0.001). In the PCP group, there was a trend toward higher maternal age (*P* = 0.07) and a higher proportion of women with spontaneous conception and spontaneous labor compared with controls (*P* = 0.07). Few participants reported drug use, all of whom also reported PAE. Cannabis use was reported by 14 women and amphetamine by 3 women. However, only 5 of these women donated placentas and 4 attended ultrasound appointments, precluding any meaningful analysis of outcomes.

#### Maternal intake of folic acid from supplements.

The National Health and Medical Research Council (NHMRC) recommends supplemental folic acid for at least 1 month prior to conception and throughout T1 of pregnancy (32). Overall, 67% of this cohort took a folic acid supplement preconception and 92% in T1 (Table 2). The proportion of women who took folic acid preconception and throughout T1 differed across control, PC, and PCP groups (*P* < 0.05), driven by a lower proportion in the PCP group compared with the PC group (*P* < 0.05). Most women (84%–85%) continued taking supplements containing folic acid throughout T2 and T3, and this was similar between control and PAE groups. Of the women who took a folic acid supplement, there were no differences between groups at any time point in the proportion with an average daily intake between the recommended 400 μg/d and the upper tolerable limit of 1,000 μg/d or in the proportions taking more or less than this range.

#### Maternal intake of folate and choline from dietary sources.

Among women who completed the dietary survey (*n* = 346), mean intake of folate was 483 μg/d (SEM = 9.6), with 20.5% of women meeting the recommended daily intake (RDI) of 600 μg/d (Supplemental Table 2). Mean intake of choline was 371 mg/d (SEM = 6.7), and 29% of women met guidelines for adequate intake (AI) of 440 mg/d. Mean intakes and proportions of women meeting guidelines did not differ between control and PAE groups.

#### Maternal plasma folate and choline concentration.

Mean maternal plasma folate concentration at 28 weeks of gestation (*n* = 225) was 40.6 nM (SEM = 1.3) (Supplemental Table 3). Using World Health Organization categories for plasma folate (33), 5 women (2.2%) were in the “possible deficiency” category (<13.5 nM), 145 women (64.5%) were in the “normal” category (13.5–45.3 nM), and 75 women (33.3%) were in the “elevated” category (>45.3 nM). At 36 weeks of gestation (*n* = 178), mean plasma folate concentration was 63.4 nM (SEM = 2.3). There were 3 women (1.7%) in the possible deficiency category, 53 (29.8%) in the normal category, and 122 (68.5%) in the elevated category. Mean folate concentrations and proportions of women within each category did not differ between control and alcohol groups. Mean maternal plasma choline concentration at 28 weeks was 10.7 μM (SEM = 0.26) and at 36 weeks was 10.1 μM (SEM = 0.21). Reference values for plasma choline have yet to be established; however, these concentrations are consistent with previous studies (34, 35) and did not differ between control and alcohol groups.

#### Association between folate and choline intake and plasma concentration.

Total intake of folate from the diet (including fortified foods) and from supplements was calculated as dietary folate equivalent (DFE). There was a moderate correlation between DFE and plasma folate at both 28 weeks (Spearman’s rho = 0.34, *P* < 0.0001, *n* = 176) and 36 weeks (rho = 0.35, *P* < 0.0001, *n* = 133). However, when divided by group, at 28 weeks there was no correlation in the control group (rho = 0.24, *P* = 0.13, *n* = 41) but a moderate correlation in the PC group (rho = 0.4, *P* = 0.0005, *n* = 75) and PCP group (rho = 0.33, *P* = 0.01, *n* = 60). This was similar at 36 weeks (Con rho = 0.25, *P* = 0.17, *n* = 30; PC rho = 0.41, *P* = 0.001, *n* = 59; PCP rho = 0.38, *P* = 0.01, *n* = 44). There was no correlation between dietary choline intake and plasma choline levels at 28 weeks overall (rho = –0.04, *P* = 0.6, *n* = 173) or when divided by group. At 36 weeks there was a weak correlation overall (rho = 0.26, *P* = 0.003, *n* = 132), driven by a moderate correlation in the PC group (rho = 0.32, *P* = 0.01, *n* = 57), with no correlation in control (rho = 0.20, *P* = 0.28, *n* = 31) or PCP groups (rho = 0.23, *P* = 0.14, *n* = 44).

### Infant data

Overall, mean gestation length for both male and female fetuses was 39 weeks (273–279 days), with few preterm births among either male (5%) or female (3%) fetuses (Table 3). Mean birth weight was in the 52nd-centile, with birthweight <10th-centile occurring in 7% of male and 7.5% of female fetuses. There were no differences between control and PAE groups in any birth measures, although there were trends toward increased gestation length (*P* = 0.06) and birth weight (*P* = 0.07) in female fetuses from the PAE group compared with controls.

### Feto-placental blood flow

**Figure 2:**
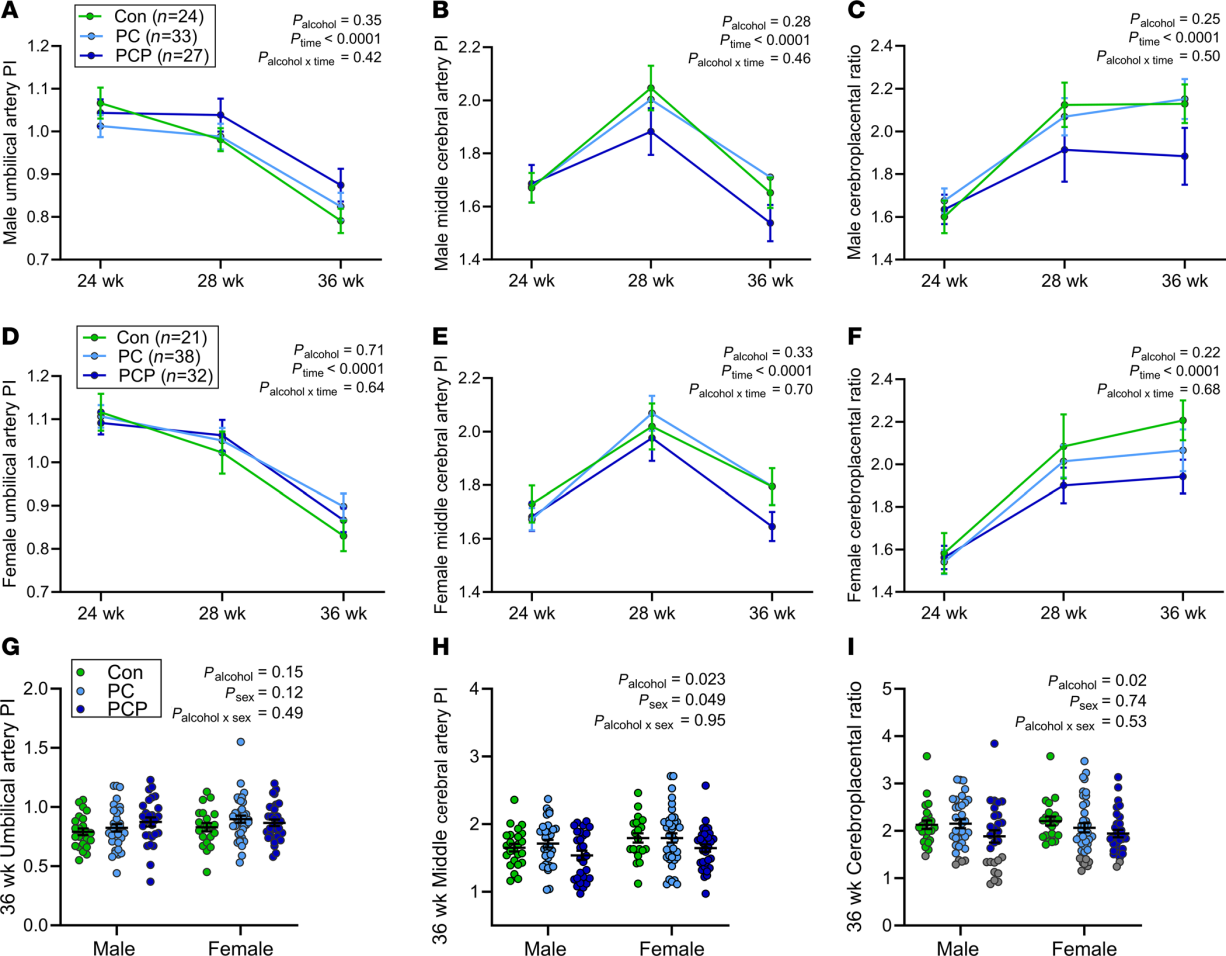
Doppler ultrasound measurements across gestation in a general pregnancy cohort. Measurements of umbilical artery pulsatility index (UAPI) and fetal middle cerebral artery pulsatility index (MCAPI) and cerebroplacental ratio across gestation (24, 28, and 36 weeks) in male (**A**–**C**) and female (**D**–**F**) fetuses and at 36 weeks’ gestation in male and female fetuses (**G**–**I**). Women were grouped by alcohol consumption into abstinent (con, green: male *n* = 24, female *n* = 21), alcohol preconception only (PC, light blue: male *n* = 33, female *n* = 38), or alcohol preconception and during pregnancy (PCP, dark blue: male *n* = 27, female *n* = 21). Data expressed as mean ± SEM. Measurements compared between groups over time (**A**–**F**), using a mixed effects model, and at 36 weeks (**G**–**I**), using 2-way ANOVA and Tukey’s post hoc test. Residuals were normally distributed. *P* < 0.05 was considered statistically significant. Fetuses with CPR <5th-centile indicated by gray data points (**I**). Complete data at 36 weeks. Missing UAPI and/or MCAPI for males at 24 and 28 weeks con (*n* = 2), PC (*n* = 1), and 28 weeks PCP (*n* = 1) and for females at 24 and 28 weeks con (*n* = 2), 24 weeks PC (*n* = 4), and 28 weeks PC (*n* = 1).

Doppler ultrasound measurements taken at 24, 28, and 36 weeks of gestation (*n* = 175) were analyzed over time between control and alcohol groups for males and females. As expected, the umbilical artery pulsatility index (UAPI) decreased over pregnancy (Figure 2, A and D), and the middle cerebral artery pulsatility index (MCAPI) increased between T2 and T3, then declined toward term (Figure 2, B and E). The ratio of the MCAPI to the UAPI, known as the CPR, indicates feto-placental blood flow, and increased over gestation (Figure 2, C and F). Thus, time was the major determinant of all measures in males and females, regardless of control or alcohol group (*P* < 0.0001). However, analysis at each time point, with alcohol and sex as factors, found that while there was no main effect of alcohol group on any measurement at either 24 or 28 weeks of gestation (Supplemental Figure 1), at 36 weeks there was a significant difference across control and alcohol groups for both the MCAPI and the CPR (*P* = 0.02, Figure 2, H and I), with no difference in UAPI (Figure 2G). Post hoc tests indicated that for MCAPI this was driven by a lower mean value in the PCP group versus the PC group (*P* = 0.02) and for CPR by a lower mean ratio in the PCP group versus the control group (*P* = 0.02). Since the CPR is more sensitive to fetal hypoxia than its individual components (36), we further investigated the CPR using linear regression. Importantly, no other maternal factors were associated with the CPR, including those listed in Table 1, as well as gestation length and maternal intake/plasma measures of folate/choline, though there was a trend toward increased CPR in women with parity of 2+ (*P* = 0.06) (Supplemental Table 4).

#### Association between PAE and clinical centiles for CPR at 36 weeks of gestation.

A CPR <5th-centile is proposed to indicate redistribution of blood flow to the fetal brain (brain sparing) in response to placental insufficiency, even in cases where the UAPI is apparently normal and fetal growth is appropriate for gestational age (AGA) (37, 38). In this cohort there were 24 pregnancies with a CPR <5th-centile (gray data points, Figure 2I), 23 from the PAE group (18%) and 1 control (2%). Fifteen of the 24 women (63%) were carrying a male fetus (1 control, 4 PC, 10 PCP), and 9 (37%) were carrying a female fetus (7 PC, 2 PCP). Although males were overrepresented, this was not statistically significant (*P* = 0.18). However, the 5 lowest CPR values were all in male fetuses (PCP group). To determine whether any maternal factors other than PAE may be risk factors for a CPR <5th-centile, the maternal characteristics in Table 1, as well as gestation length and maternal intake/plasma measures of folate and choline, were compared between women with a CPR above or below the 5th-centile. Among women with a CPR <5th-centile, there were trends toward a lower proportion who took folic acid preconception (*P* = 0.05) and a higher proportion who were nulliparous (*P* = 0.08) or had assisted conception (*P* = 0.07) (Supplemental Table 5).

Logistic regression analysis predicted that PC was associated with an 8.1-fold increase in the likelihood of a CPR <5th-centile compared with the control group (95% CI, 1.0–64.8, *P* = 0.05), and PCP was associated with an 11.2-fold increase (95% CI 1.4–90.0, *P* = 0.02) (Supplemental Table 6). There was a trend toward an increased likelihood of CPR <5th-centile in women with a parity of 0 compared with 1 or more (OR 2.3, 95% CI 0.93–5.50, *P* = 0.071), women who did not take folic acid supplements preconception (OR 2.3, 95% CI 0.97–5.54, *P* = 0.059), or women who had assisted conception (OR 3.5, 95% CI 0.96–12.6, *P* = 0.058). Adjusted analysis was not performed because of the small number of women with CPR <5th-centile. Given that a low CPR is associated with placental insufficiency, we next compared placental growth measurements and growth factor expression between control and alcohol groups for male and female placentas.

### Placental data

#### Placental weight and dimensions at delivery.

There were no differences in mean placental weight between control and PAE groups in either sex. However, while in the control group, male placentas weighed on average 50 g more than female placentas (*P* = 0.04, Table 4) consistent with previous reports (39), in the PAE groups, there was no difference between male and female placenta weight. Male PAE placentas weighed 25–27 g less on average than male control placentas, whereas female PAE placentas weighed 27–35 g more than female control placentas. There were no differences in birth weight/placenta weight ratio or in placenta length or width between groups in either sex. However, male PAE placentas had reduced thickness compared with control placentas (*P* = 0.03, Table 4). This finding was supported by measurements of placental thickness using ultrasound images collected a few weeks earlier at 36 weeks of gestation, which identified a trend toward reduced mean thickness in the male PCP placentas compared with control placentas (*P* = 0.066, Supplemental Figure 2) and no differences in female placentas. For pooled male and female data, there was a moderate correlation between thickness measurements from ultrasound images at 36 weeks and measurements of placental thickness at delivery (rho = 0.35, *P* < 0.001, *n* = 144).

#### Placental expression of angiogenic growth factors and receptors.

**Figure 3:**
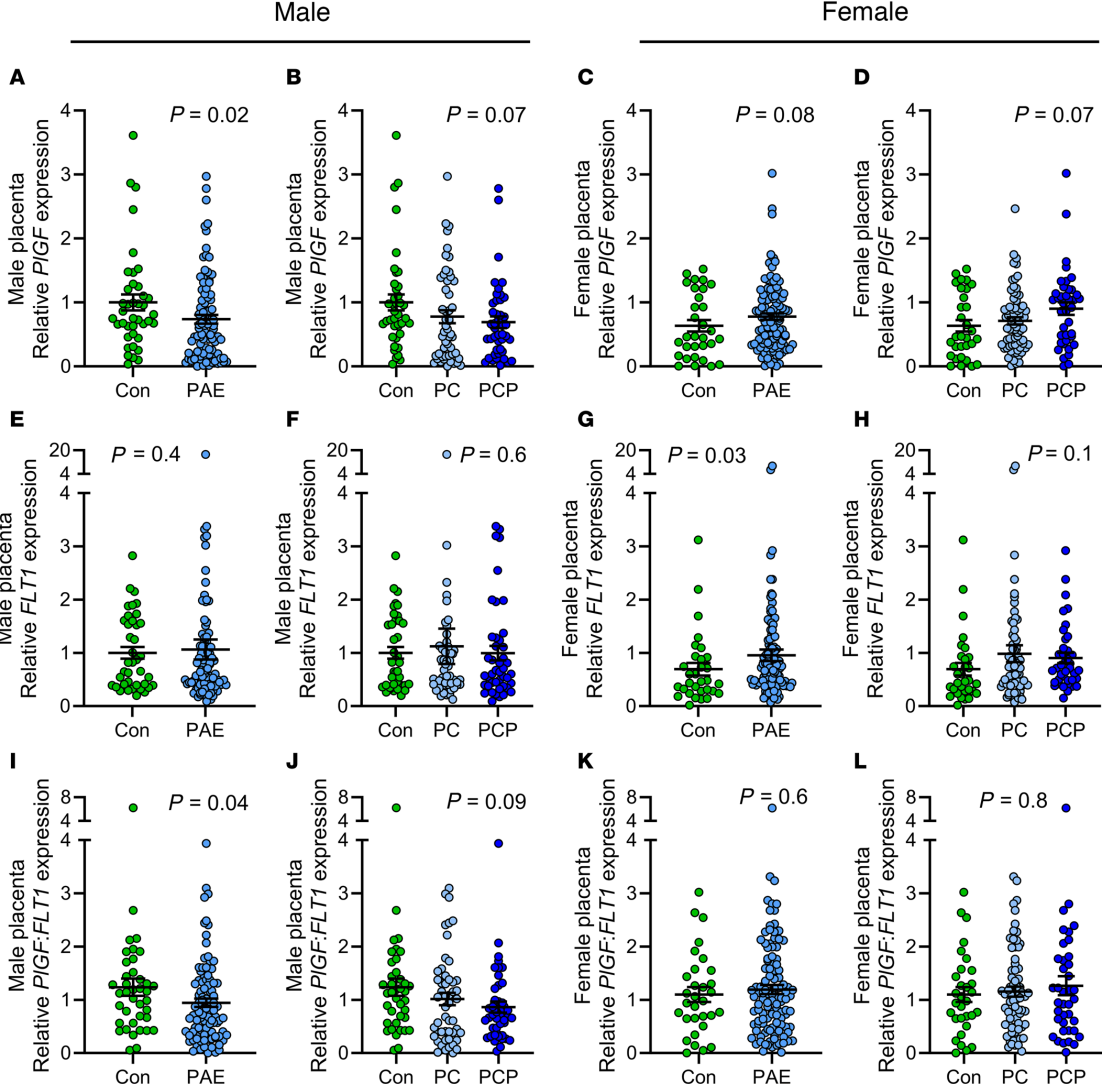
Placental expression of growth factors by alcohol and sex. Expression of placental growth factor (*PlGF*) (**A**–**D**), VEGF receptor 1 (*FLT1*) (**E**–**H**), and *PlGF/FLT1* (**I**–**L**). Placentas collected from abstinent women (con: male *n* = 40, female con *n* = 31) and women reporting prenatal alcohol exposure (PAE) (male *n* = 95, female *n* = 112). The PAE group was further divided by timing into preconception only (PC: male *n* = 51, female *n* = 73) or preconception and during pregnancy (PCP: male *n* = 44, female *n* = 39). Gene expression normalized to β-actin (*ACTB*), and fold-change expressed relative to male con group. Data expressed as mean ± SEM. Con and PAE groups compared using unpaired *t* test/1-way ANOVA where residuals were normally distributed or where transformation normalized distribution. Otherwise, Mann-Whitney/Kruskal-Wallis tests were used (**E**, **F**, **H**, and **J**). *P* < 0.05 was considered statistically significant, and *P* = 0.05–0.1 was considered a trend. *FLT1* was below detection limit for 1 male control, as was *PlGF* for 1 female PC.

Compared with male control placentas, male PAE placentas had reduced expression of *PlGF* mRNA (Figure 3, A and B), no change in *FLT1* (Figure 3, E and F), and a reduction in the ratio of *PlGF/FLT1* (Figure 3, I and J). In female PAE placentas, there was a trend toward an increase in *PlGF* (Figure 3, C and D), an increase in *FLT1* (Figure 3, G and H), and no change in the ratio of *PlGF/FLT1* (Figure 3, K and L), compared with controls. Similar to the findings for placental weight, male control placentas had higher expression of both *PlGF* and *FLT1* compared with female control placentas (*P* = 0.02) whereas there were no sex differences in the PAE group (Supplemental Figure 3). Among control placentas there were no sex differences in the *PlGF/FLT1* ratio, whereas among PAE placentas females had a higher ratio compared with male placentas. There were no differences in expression of *VEGFA* or *KDR* between groups or sexes (Supplemental Figure 4), though there was a trend toward reduced *VEGFA* in male PAE placentas compared with controls (*P* = 0.06). Since epigenetic regulation has been identified as a mechanism through which PAE alters gene expression, we next measured placental global DNAm.

#### Placental global DNAm.

Linear regression analyses were used to investigate associations between DNAm and maternal factors after adjustment for assay plate number (Supplemental Table 7). Potential predictors of male placental global DNAm at *P* < 0.1 were PCP group (*P* = 0.05), pregnancy complications (*P* = 0.04), and T3 folic acid supplementation (*P* = 0.06). In adjusted analysis, there was a decrease in mean DNAm in the male PCP group compared with the control group (*P* = 0.048, Table 5). Potential predictors of female placental global DNAm at *P* < 0.1 were PC group (*P* = 0.09), parity (*P* = 0.09), and Caucasian race (*P* = 0.02). In adjusted analysis, only Caucasian race and parity were significantly associated with increased DNAm (*P* < 0.05). Since these data provided weak evidence of an association between PAE and altered global DNAm, we next investigated whether this was supported by any associations between PAE and expression of *DNMT*s or levels of key methyl donors.

#### Expression of DNMT enzymes in the placenta.

**Figure 4:**
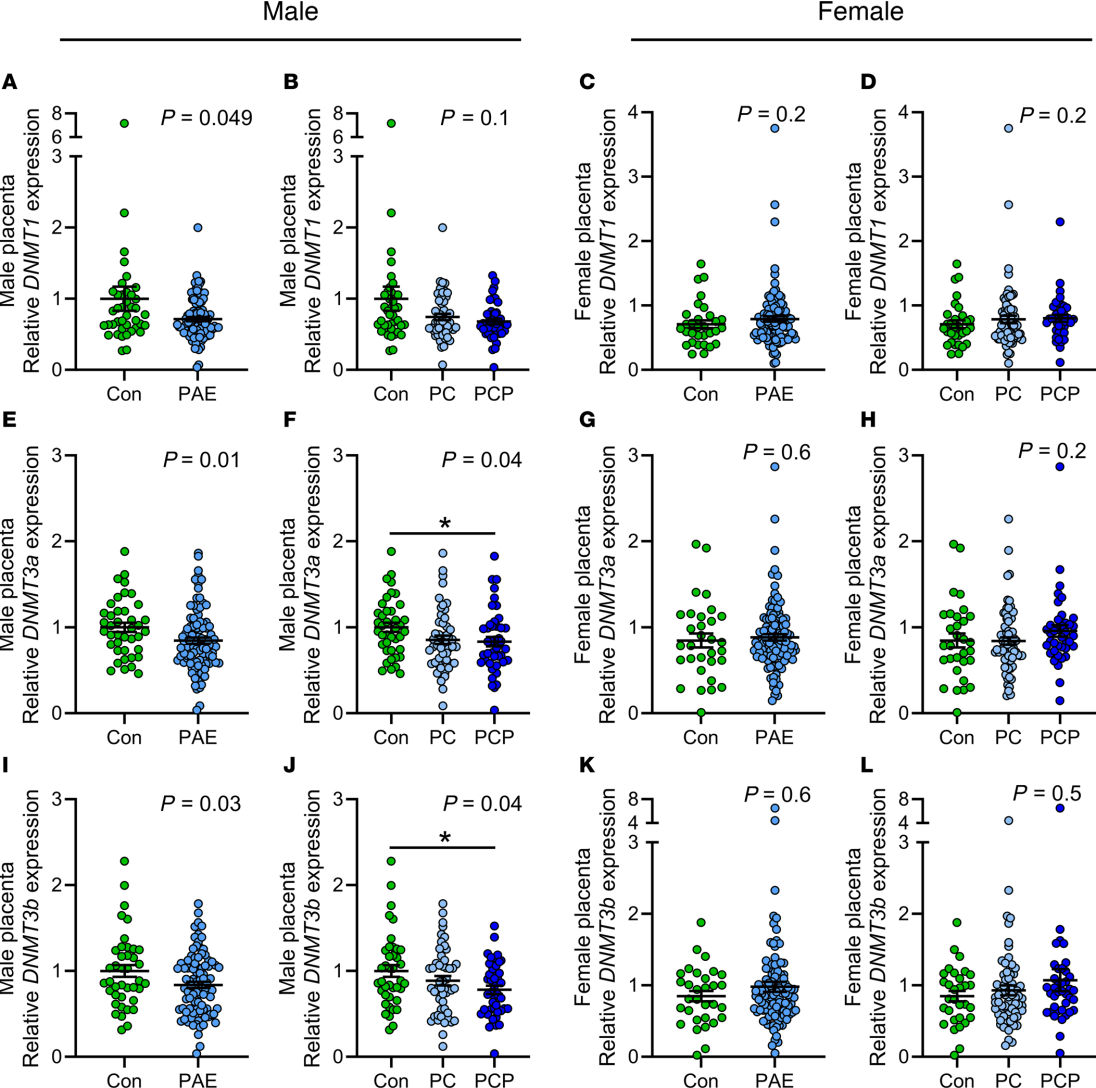
Placental expression of *DNMT*s by alcohol and sex. Expression of DNA methyltransferases *DNMT1* (**A**–**D**), *DNMT3a* (**E**–**H**), and *DNMT3b* (**I**–**L**). Placentas collected from abstinent women (con: male *n* = 40, female *n* = 31) and women reporting prenatal alcohol exposure (PAE) (male *n* = 95, female *n* = 112). The PAE group was also divided by timing into preconception only (PC: male *n* = 51, female *n* = 73) or preconception and during pregnancy (PCP: male *n* = 44, female *n* = 39). Gene expression normalized to *ACTB*, and fold-change expressed relative to the male con group. Data expressed as mean ± SEM. Con and PAE groups compared using unpaired *t* test/1-way ANOVA where residuals were normally distributed or where transformation normalized distribution (**I** and **J**). Otherwise, Mann-Whitney/Kruskal-Wallis tests were used. *P* < 0.05 was considered statistically significant, and *P* = 0.05–0.1 was considered a trend. *P < 0.05 for Tukey’s multiple comparisons. *DNMT3a* was below detection limit in 1 female PC placenta, as was *DNMT3b* in 2 female PC placentas.

DNMTs catalyze the transfer of a methyl group from SAM to the DNA molecule for maintenance methylation (DNMT1) or de novo methylation (DNMT3a and 3b). In male PAE placentas compared with control placentas, mRNA expression of all 3 *DNMT*s was decreased (*P* < 0.05, Figure 4, A, E, and I). Reduced expression of *DNMT3a* and *DNMT3b* was driven by a significant reduction in the PCP group (*P* < 0.05, Figure 4, F and J), consistent with the reduced global DNAm in the PCP group. In female placentas there were no differences between groups in expression of *DNMT*s (Figure 4).

#### Quantification of 1CM components in the placenta.

**Figure 5:**
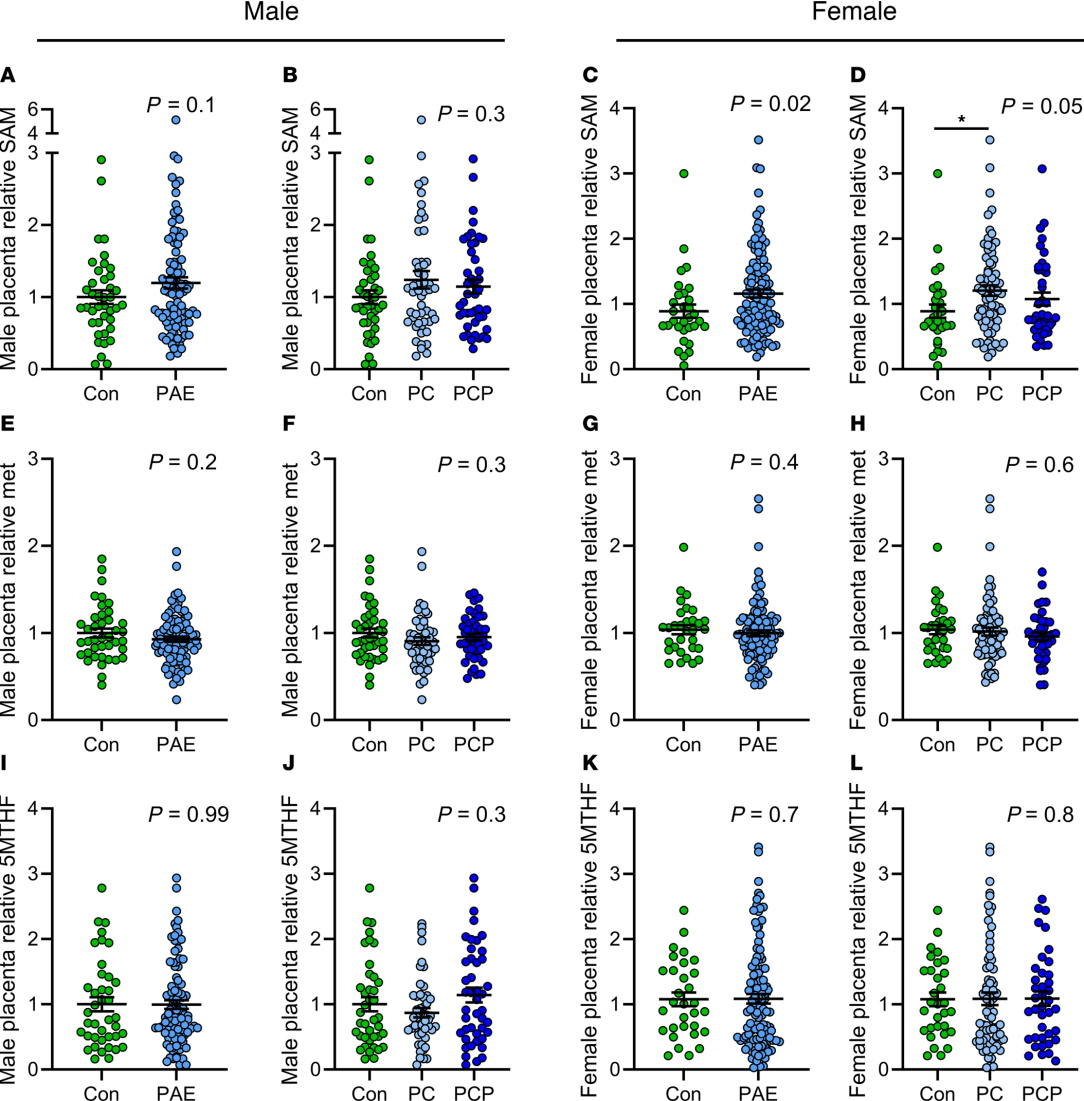
Relative levels of methyl donors in the placenta by alcohol and sex. Relative levels of *S*-adenosylmethionine (SAM) (**A**–**D**), methionine (met) (**E**–**H**), and 5-methyltetrahydrofolate (5MTHF) (**I**–**L**). Placentas collected from abstinent women (con: male *n* = 40, female *n* = 31) or women reporting prenatal alcohol exposure (PAE) (male *n* = 95, female *n* = 111). The PAE group was further divided by timing of exposure into preconception only (PC: male *n* = 51, female *n* = 72) or preconception and during pregnancy (PCP: male *n* = 44, female *n* = 39). The ratio of methyl donor/internal standard was calculated and mean fold-change ± SEM expressed relative to the male con group. Expression differences across groups compared using unpaired *t* test/1-way ANOVA where residuals were normally distributed or where transformation normalized distribution. Otherwise, Mann-Whitney/Kruskal-Wallis tests were used (**G** and **H**). *P* < 0.05 was considered statistically significant, and *P* = 0.05–0.1 was considered a trend. *P < 0.05 for Tukey’s multiple comparisons. One female PC sample failed mass spectrometry.

There were no differences in mean relative levels of SAM in male placentas (Figure 5, A and B). In female placentas there was a significant increase in SAM in the PAE group, compared with controls (*P* = 0.02, Figure 5C). Post hoc test revealed this was driven by an increase in the PC group (*P* < 0.05, Figure 5D), consistent with the trend toward increased global DNAm in the PC group (unadjusted analysis). There were no differences in the mean levels of methionine (Figure 5, E–H) or 5-methyltetrahydrofolate (5MTHF) (Figure 5, I–L) in either sex.

#### Expression of nutrient regulators in the placenta.

**Figure 6:**
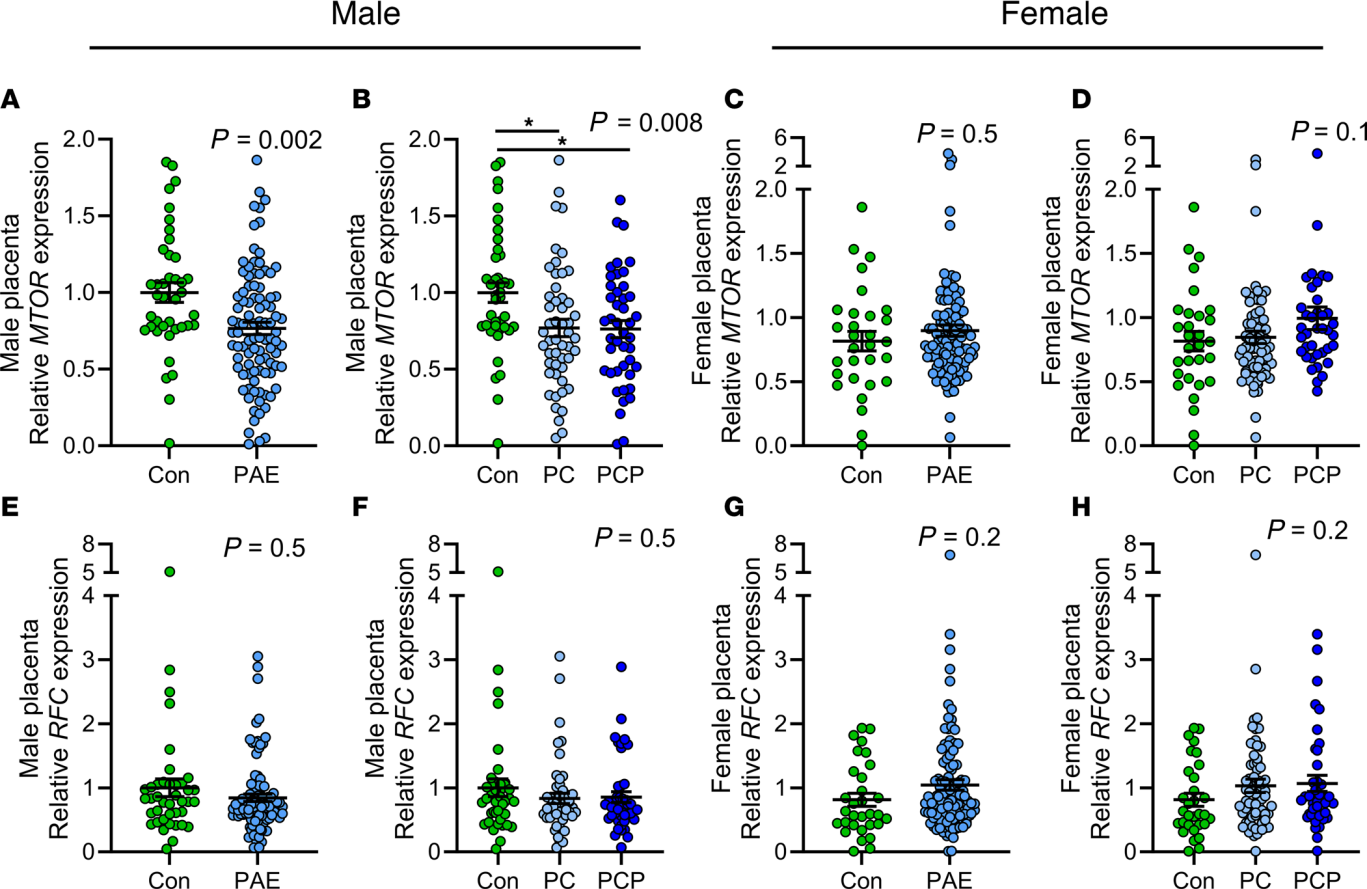
Placental expression of nutrient regulators by alcohol and sex. Expression of mechanistic target of rapamycin (*MTOR*) (**A**–**D**) and reduced folate carrier (*RFC*) (**E**–**H**). Placentas collected from abstinent women (con: male *n* = 40, female con *n* = 31) or women with prenatal alcohol exposure (PAE) (male *n* = 95, female *n* = 112). The PAE group was also divided by timing of exposure into preconception only (PC: male *n* = 51, female *n* = 73) or preconception and during pregnancy (PCP: male *n* = 44, female *n* = 39). Gene expression normalized to *ACTB*, and fold-change expressed relative to male con group. Data expressed as mean ± SEM. Con and PAE groups compared using unpaired *t* test/1-way ANOVA where residuals were normally distributed or where transformation normalized residuals. Otherwise, Mann-Whitney/Kruskal-Wallis tests were used (**C**–**H**). *P* < 0.05 was considered statistically significant, and *P* = 0.05–0.1 was considered a trend. *P < 0.05 for Tukey’s multiple comparisons test. *MTOR* was below detection limit in female con (*n* = 1), PC (*n* = 2), and PCP (*n* = 1), as was *RFC* in female PC (*n* = 2).

Expression of *MTOR* mRNA was reduced in male PAE placentas compared with controls (*P* = 0.002, Figure 6A), in both the PC and PCP groups (*P* < 0.05, Figure 6B), with no difference between groups in female placentas (*P* = 0.54, Figure 6, C and D). There were no differences in mRNA expression of *RFC* between control and PAE groups in either sex (Figure 6, E–H).

## Discussion

This study found that PAE around conception was highly prevalent among a cohort of predominantly well-educated women with planned pregnancies. There was a significant increase in the likelihood of a low CPR in women who reported PAE, which indicates fetal brain sparing because of placental insufficiency (40). Half of the women with a low CPR reported PAE only around conception (i.e., PC group), suggesting that all cases of low CPR could have originated from exposure around conception, regardless of whether alcohol was continued (i.e., PCP group). Most pregnancies with a low CPR were male fetuses, and the 5 lowest CPR values were in males, which may indicate sexually dimorphic placental adaptions to PAE. This is supported by reduced placental thickness and reduced growth factor expression in male, but not female PAE placentas, compared with controls. In addition, compared with control placentas, male PAE placentas had decreased global DNAm and decreased expression of DNMTs, whereas in female PAE placentas there was a trend toward increased DNAm and an increase in the universal methyl donor, SAM. Together, these findings may suggest an epigenetic link between PAE in early pregnancy and sexually dimorphic placental outcomes.

### Placental insufficiency may link early PAE and infant outcomes.

Placental insufficiency typically originates in early pregnancy because of shallow trophoblast invasion into the uterus and subsequent inadequate remodeling of maternal spiral arteries, which can result in failure of the placenta to deliver sufficient oxygen and nutrients to meet the demands of the growing fetus (41). This can lead to fetal hypoxia, triggering compensatory mechanisms to prioritize blood flow to the fetal brain at the expense of fetal growth (brain sparing), and depending on duration and severity, can result in FGR (42). Studies investigating the consequences of a low CPR in FGR fetuses have reported associations with poor neurodevelopmental outcomes in children (43, 44). However, in cohorts such as ours, where fetal growth is generally AGA, Doppler ultrasound assessment in late gestation is not routinely conducted. Thus, there is little information on T3 CPR and infant neurodevelopmental outcomes. However, one study of T3 AGA fetuses with brain sparing (indicated by increased frontal brain perfusion) found an association with poorer neurobehavioral scores in neonates (45). Although, to the best of our knowledge, an association between PAE and decreased CPR has not been reported previously in women, a study of first-trimester alcohol exposure in nonhuman primates found an association between PAE and increased resistance to placental blood flow using Doppler ultrasound. This was accompanied by increased frequency of placental infarctions as well as altered placental gene expression (19). An earlier study in the same model found reduced oxygen supply to the fetal vasculature and reduced brainstem and cerebellar volume in alcohol-exposed fetuses in early third trimester using in utero MRI (20). Disruption of early placental development leading to placental insufficiency and fetal brain sparing in late pregnancy may therefore be a mechanism through which PAE around conception indirectly impacts fetal brain development and neurodevelopmental outcomes in children.

### Potential risk factors for placental insufficiency.

It is well understood that PAE in some women can impact fetal development whereas a similar pattern of PAE in other women has no apparent effects. This has prompted research into maternal factors that exacerbate or protect against fetal effects of PAE (46). In the current study, while PAE was the only maternal factor significantly associated with reduced CPR (Supplemental Table 6), there was a trend toward an association between a clinically low CPR and nulliparity, lack of preconception folic acid supplementation, and the use of assisted reproduction to conceive. There were only 12 women with Doppler measures who used assisted conception in this cohort, but given that in vitro fertilization (IVF) has been found in association with ischemic placental disease (47), it is possible that assisted conception increases the risk of placental insufficiency in women with PAE. Similarly, nulliparity is a known risk factor for complications related to poor placental development, including preeclampsia and stillbirth (48, 49), which have also been found in association with a low CPR (50, 51). However, the potential association between lack of preconception folic acid and low CPR is of particular interest, as this is a modifiable risk factor. Folate is essential for early embryo development, as evidenced by the use of the folate analog, methotrexate, to terminate ectopic pregnancies (52). Folate supplies methyl groups for the generation of nucleotides required for DNA replication during cell division and for DNAm. A folate-deficient diet in pregnant mice results in reduced placental trophoblast invasion into the uterus, altered placental morphology, and placental abruption (53). Culture of human placental cell lines in reduced-folate medium decreases trophoblast viability, alters DNAm, and reduces expression of growth factors and matrix metalloproteases, which are important for trophoblast invasion (54, 55). Alcohol is well-known to reduce absorption of folate and availability of folate-derived methyl groups (25). Thus, a lack of preconception folic acid supplementation may increase vulnerability to the effects of PAE on folate levels, resulting in altered epigenetic regulation of gene expression and deficient placentation.

### Timing of PAE and sex-specific placental outcomes.

Prior studies have found that PAE in women either reduces placenta weight (17, 56) or has no effect, as reported in the current study and others (18, 57). This may be due to differences in quantity, frequency, and timing of PAE, though this is difficult to determine because of the different methods of collecting and categorizing alcohol data across studies, as discussed in our systematic review (16). However, the current study found that PAE was associated with sex-specific changes in placental growth measures that may depend upon the timing of exposure, factors that have not been widely investigated in women. We found that male PAE placentas had reduced mean thickness, reduced expression of *PlGF*, and reduced *PlGF/FLT1* ratio compared with controls, suggesting an antiangiogenic state. This is consistent with the lower expression of *MTOR*, which indicates nutrient deficiency and a state of reduced growth (58). Reduced placental expression of PlGF has been found previously in women with PAE in association with reduced expansion of placental blood vessel area from midgestation to term, which also correlated with impaired cortical vessel organization in fetal brains (17). This suggests that PAE-induced downregulation of PlGF in the placenta can impact vasculature in both the placenta and fetal brain. In the current study DNAm and expression of *DNMT*s were also reduced in male placentas, suggesting the involvement of epigenetic mechanisms. Many of these changes were most apparent in placentas from pregnancies with alcohol exposure beyond the periconception period (i.e., the PCP group). Similarly, most of the male fetuses with a low CPR (71%) were from the PCP group. In contrast, among female PAE placentas, there was a trend toward increased DNAm (unadjusted analysis), and an increase in SAM, predominantly in the PC group. Most female fetuses with a low CPR (78%) were also from the PC group.

Sexually dimorphic placental adaptions depending on timing of exposure have been identified in response to other in utero perturbations and suggest that females are often more vulnerable to exposures in early pregnancy whereas males are more vulnerable to exposures in later pregnancy (reviewed in refs. 59, 60). A large study that examined sex ratio throughout gestation found that loss of viability was more prevalent in female embryos and fetuses prior to 20 weeks of gestation, whereas loss of male fetuses generally occurred after 20 weeks and at a lower rate than female losses in early pregnancy (61). Studies of IVF pregnancies also reveal a skewed sex ratio of live birth in favor of males (62), and studies in mice have identified impaired X chromosome inactivation (XCI) in female embryos as a potential mechanism (63). Similarly, we have previously reported that in our rat model, PAE around conception, but not beyond, resulted in female-specific reductions in placental vasculature and placental volume along with reduced expression of *Xist* and *Rlim* (initiators of XCI), indicating that impaired XCI may also be a mechanism of alcohol-induced placental defects (13).

Furthermore, the current study also found sex differences in placental weight and growth factor expression in control placentas, as previously documented (39, 64), which were not present in PAE placentas. A loss of sex differences in the placenta has been proposed to be an important indicator of sex-specific placental adaption to a stressful environment and has been identified in pregnancies complicated by intrauterine growth restriction and maternal obesity (65, 66). Taken together, these findings suggest sexually dimorphic placental responses to PAE, which may depend upon timing of exposure, emphasizing the importance of examining placental outcomes separately by sex of the placenta and timing of PAE.

### Maternal folate and choline.

Insufficient intake of micronutrients among women with PAE has been suggested as a risk factor for FASD (46). In the current study we focused on the micronutrients folate and choline because of their roles as methyl donors for DNAm and therefore their potential to ameliorate the effects of PAE. Although there were no differences between control and PAE groups in intake of folate from dietary sources, few women overall met the RDI for folate from foods, indicating a high dependence on supplements. However, only half of women overall took the recommended folic acid supplementation preconception, and a lower proportion of women in the PAE group took supplemental folic acid preconception and T1. As discussed above, this may increase vulnerability to the negative effects of PAE on folate levels, which can impair placentation and result in placental insufficiency. Although samples were not available to measure folate in early pregnancy, it was interesting that plasma folate measurements at 28 and 36 weeks did not correlate with folate intake in the control group but were moderately correlated with intake in the PAE groups. Similarly, plasma choline concentration correlated with choline intake in the PC group, but not control or PCP groups, perhaps suggesting altered nutrient transport or metabolism between groups, although this requires further investigation. Unlike folate, choline is generally not included in prenatal supplements and must be obtained from foods. Few women in this study met the AI for choline, consistent with other pregnancy cohorts in Australia (67, 68). Therefore, any protective effects of increased choline intake in women with PAE could not be assessed in this cohort. In fact, choline supplementation studies in pregnant women suggest that more than double the AI (980 mg/d) may be required to meet additional demands during pregnancy (69). Women with PAE may require an even higher level of choline supplementation to protect against fetal harm as suggested by the findings from 2 clinical trials of choline supplementation in women with PAE. One trial found that choline supplementation at 2,000 mg/d improved infant growth and cognitive function at 12 months (70), whereas the other trial found that choline supplementation at 750 mg/d had no beneficial effects on cognitive function at 6 months (71).

### Limitations.

The main limitation of this study is that participants were not asked about amount of alcohol consumed. Therefore, a dose-response relationship could not be investigated. However, drinking patterns are likely comparable to a uniquely detailed study of alcohol consumption in a similarly well-educated cohort in Australia, the AQUA cohort, which reported moderate consumption with some binge episodes usually prior to pregnancy awareness (6). It is also important to note that we cannot be certain that male and female fetuses/placentas received similar exposure to alcohol overall. Therefore, while this study identified apparent sex-specific alterations associated with PAE, interaction effects were not tested. We also acknowledge that the placenta contains heterogenous cell types, which may vary with respect to DNAm and gene expression. Therefore, it is possible that PAE-associated alterations in these measures could be underestimated because of opposing effects in different cell types. Indeed, we have previously demonstrated differential effects on gene expression in our animal model when different parts of the placenta (junctional and labyrinth zones) were examined separately (10). Another limitation was variation in sample sizes for different measurements, which was largely because of the impact of COVID-19 restrictions on participant appointments and sample collection. Nonfasted plasma samples were used for measurements of choline and folate, which may only reflect recent intake. Finally, although a broad range of maternal factors were investigated for their relationships with alcohol consumption and outcome measures, there may be other potential confounders that have not been accounted for in the analyses.

### Conclusions.

PAE around conception in women is associated with increased likelihood of a low CPR in late gestation, particularly in males, which may have implications for fetal brain development. PAE was associated with sex-specific alterations in placental growth measures, DNAm, and gene expression, suggesting epigenetic alterations as an underlying mechanism. Low intake of folate and choline around conception may increase vulnerability to the effects of PAE on DNAm. These findings highlight the need to increase awareness of the importance of meeting nutritional guidelines for women of reproductive age and of the potentially harmful effects of PAE around conception as well as during pregnancy.

## Methods

All data and sample collection was performed blinded to exposure. Additional details are in Supplemental Methods.

### Sex as a biological variable

This study examined associations between PAE in women and measurements in male and female feto-placental units and reports sexually dimorphic outcomes.

### Study participants

The QFC pilot study was conducted at Mater Mothers’ Hospital, Brisbane, Queensland, Australia, from 2018 through 2021, with prospective recruitment of 472 women at 12–24 weeks’ gestation. A detailed study protocol has been published (72). Briefly, all pregnant women from private and public obstetric services were offered the opportunity to enroll, and only women who were unable to provide informed consent were excluded. Inclusion criteria for this substudy were singleton pregnancy and information on alcohol consumption.

#### Alcohol and drug use.

At enrollment, a research midwife collected information on alcohol consumption and cannabis and amphetamine use in the 12 weeks preconception, in weeks 1–12 of pregnancy, or during the previous 2 weeks. Use in the previous 2 weeks was collected again at 24, 28, and 36 weeks of gestation. At 24 weeks, women completed the Australian Eating Survey (AES) (73), which includes questions on typical alcohol consumption over the previous 6 months. Participants answering no to all alcohol questions were assigned to the control group (con, *n* = 109) while those answering yes to any alcohol question were assigned to the PAE group (PAE, *n* = 302). The PAE group was also divided into women who reported alcohol only in the 12 weeks preconception (PC group, *n* = 174) and women who also reported alcohol at any time during pregnancy (PCP group, *n* = 128; Supplemental Table 1).

#### Maternal folate and choline intake.

Folic acid intake from vitamin supplements (average μg/d) was determined from the folic acid content (μg) of the brand(s) of vitamin(s) reported and frequency of use over 1 week. Intake of folate and choline from foods was calculated using the AES (73), which participants completed at 24 weeks of gestation, detailing typical intake of 120 food and beverage items over the previous 6 months. Standard portion sizes were derived from the National Nutrition Survey (74). DFE, which includes natural folate content and folic acid fortification for each item, was determined using the AUSNUT 2011–2013 database (75). This database has been expanded to include choline (76), allowing estimation of dietary choline intake.

#### Plasma choline and folate concentration.

Plasma choline concentration was measured using liquid chromatography–tandem mass spectrometry (LC-MS/MS). Pooled dialyzed plasma was spiked with choline to create a standard curve as previously described (77). Standards/samples were precipitated with acetonitrile containing d9-choline internal standard (10 μM final). Chromatographic separation was achieved using a C18 column (Kinetex 1.7 μm, 10 × 2.1 mm, 100 Å, Phenomenex), and analyses were carried out on a QTRAP 5500 mass spectrometer (AB SCIEX) using electrospray ionization in positive-ion mode with multiple reaction monitoring of *m*/*z* transitions: choline 104.2→60.0, d9-choline 113.1→69.1. Peak integration was performed using MultiQuant software v2.0 (AB SCIEX). The peak area ratio of choline to internal standard was calculated for all standards/samples.

Plasma folate concentration was measured using the Cobas e411 immunoanalyzer (Roche Diagnostics), with assay reagents from the same supplier: Elecsys Folate III assay (7559992190), calibrator set (7560001190), and Diluent Universal (5192943190). Plasma samples were vortexed, centrifuged briefly, and transferred to a clean tube for measurement.

#### Doppler ultrasound measurements.

Doppler ultrasound measurements were collected by a trained sonographer at 24, 28, and 36 weeks of gestation as previously described (78). The UAPI and the fetal MCAPI were recorded, and the CPR was calculated by dividing MCAPI by UAPI. CPR <5th-centile at 36 weeks was determined using sex-specific centiles (male: 1.47, female: 1.42) (79). Ultrasound images of the placenta at 36 weeks allowed calculation of mean placental thickness using measurements taken at 3 regions (cord insertion, mid/thickest, edge).

### Infant data

Data on gestation length (days), infant birth weight (g), head circumference, and length (cm) were collected by medical staff at delivery. Birth centile was calculated using Perinatal Institute Gestation Network centile calculator (Grow v8.0.6.1 2020), which adjusts for birth weight, sex, gestation length, and maternal ethnicity, height, weight, and parity.

### Placenta data

Placentas were collected within 2 hours of delivery using standard methodology (80). Measurements of placenta length (cm), width (cm), thickness (cm), and weight (g) were recorded. Eight full-thickness samples of villous tissue (~1 cm^3^) were dissected, taking 2 samples from each quadrant, midway between cord insertion (or center), and edge of the placenta. The upper ~2 mm of basal tissue was trimmed away, and samples were rinsed in saline, finely minced and combined into a single homogenous sample, distributed into cryovials, snap-frozen, and stored at –80°C. For subsequent analyses, frozen placenta samples (*n* = 278) were finely crushed under liquid nitrogen and homogenized using a FastPrep-5G bead homogenizer (MP Biomedicals).

#### Measurement of methyl donors.

Crushed placental tissue was weighed and homogenized in 3 volumes of milliQ water (MilliporeSigma). Sample preparation and LC-MS/MS were conducted as described for plasma samples above, with multiple reaction monitoring of *m*/*z* transitions: d9-choline 113.1→69.1, 5MTHF 460.2→313.1, methionine 150.0→104.2, homocysteine 136.0→90.2, SAM 399.2→250.0, *S*-adenosylhomocysteine (SAH) 385.2→88.0. Peaks for homocysteine and SAH were identified in only a small number of samples and therefore not included in further analysis. Peak area ratio of each molecule to internal standard was calculated.

#### Gene expression.

RNA was extracted from 30 mg of homogenized placental tissue using RNeasy extraction kits (74106, QIAGEN) as per manufacturer’s instructions. DNase treatment and cDNA synthesis were performed using the QuantiTect reverse transcription kit as per manufacturers’ instructions (205311, QIAGEN). Negative (-RT) reactions were included for a random subset of samples. Gene expression was measured using quantitative PCR on an Applied Biosystems Quantstudio 6 Flex Real-Time PCR System (Thermo Fisher Scientific). Each 10 μL reaction contained 10 ng cDNA, QuantiNova SYBR Green PCR Master Mix (QIAGEN), and KiCqStart SYBR Green primers (MilliporeSigma) (Supplemental Table 8). Relative gene expression was determined using the comparative threshold method (ΔΔCt) and normalized to β-actin (*ACTB*), which was stable across groups and sexes. A pooled sample of all cDNAs was repeated in triplicate, allowing calculation of the coefficient of variation (<2% for all genes).

#### Global DNAm.

Genomic DNA was extracted from 20 mg of crushed homogenized placental tissue using the DNeasy kit (69506, QIAGEN) as per manufacturer’s instructions. Concentration/purity was measured using a NanoDrop 2000 spectrophotometer (Thermo Fisher Scientific) and a portion of the sample further diluted to 50 ng/μL (±2 ng/μL). The 260:280 ratio for all samples was greater than 1.8. Methylation of cytosine residues was quantified using a global DNAm assay as per manufacturer’s instructions (ab233486, Abcam) and expressed as percentage of total DNA.

### Statistics

For categorical variables, differences between control and PAE group(s) were analyzed using Fisher’s exact/χ^2^ test. For continuous variables, differences between control and PAE group(s) were assessed using unpaired 2-tailed *t* test and ANOVA with Tukey’s multiple comparisons test. Figure 2 shows 2-way ANOVA. Figures 3–6 show 1-way ANOVA. Residuals were assessed for normality using D’Agostino-Pearson, Anderson-Darling, and Shapiro-Wilk tests and variances assessed using *F* test or Brown-Forsythe and Bartlett’s tests. Where residuals were non-normally distributed, data were transformed to achieve normal residuals (see Supporting Data Values), or otherwise nonparametric Mann-Whitney *U* test or Kruskal-Wallis test with Dunn’s multiple comparisons test were used. Nontransformed values are plotted on graphs. Doppler measures compared between control and alcohol groups over time using a mixed effects model or at a single time point using 2-way ANOVA with alcohol and sex as factors and Tukey’s multiple comparisons test. Above analyses were conducted using GraphPad Prism (v9.4.1). Correlation/regression analyses were conducted using STATA/SE 17. Spearman’s rank correlation was used for non-normally distributed data. For linear regression analyses, distribution of residuals was visually inspected for normality using diagnostic distributional plots. Univariate analysis was used to determine potential predictors at *P* < 0.1 for inclusion in multivariate analyses. For all analyses *P* < 0.05 was considered statistically significant and *P* = 0.05–0.1 considered a trend.

### Study approval

The study was approved by Mater Misericordiae Ltd Human Research Ethics Committee (HREC/MML/61799), ratified by The University of Queensland Human Research Ethics Committee (2020001492). The study was conducted according to the Declaration of Helsinki principles, and written informed consent was given by all participants before enrollment.

### Data availability

The full data set cannot be made publicly available, as the detailed nature of the data may allow identification of individual participants. Some data may be available upon request. Data underlying figures and tables (except maternal/infant characteristics as per above) can be found in the Supporting Data Values file.

## Author contributions

SES contributed to study design, sample collection, experimental analyses, data analysis, and writing of the manuscript. LKA, JSMC, LAG, KMM, and VLC contributed to study design and data analysis. CE and EC contributed to data collection. JMK and CV contributed to sample collection and experimental analyses. SES, CE, EC, CV, JMK, LKA, JSMC, LAG, KMM, and VLC critically revised the manuscript.

## Supplementary Material

Supplemental data

ICMJE disclosure forms

Supporting data values

## Figures and Tables

**Table 1:**
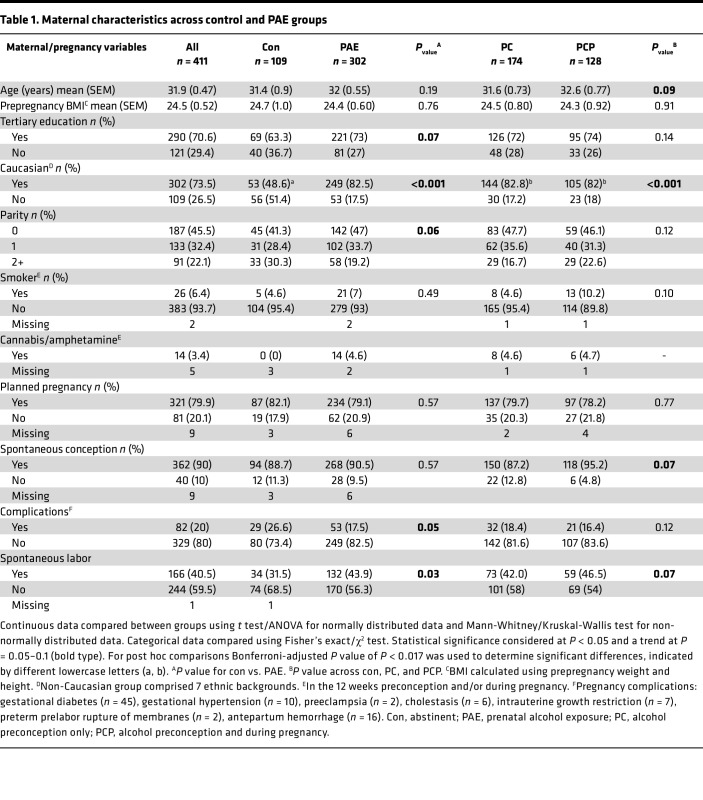
Maternal characteristics across control and PAE groups

**Table 2:**
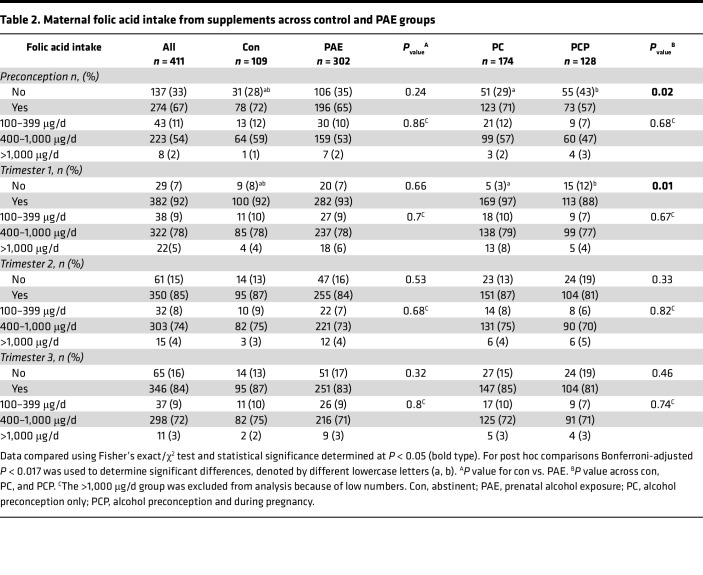
Maternal folic acid intake from supplements across control and PAE groups

**Table 3:**
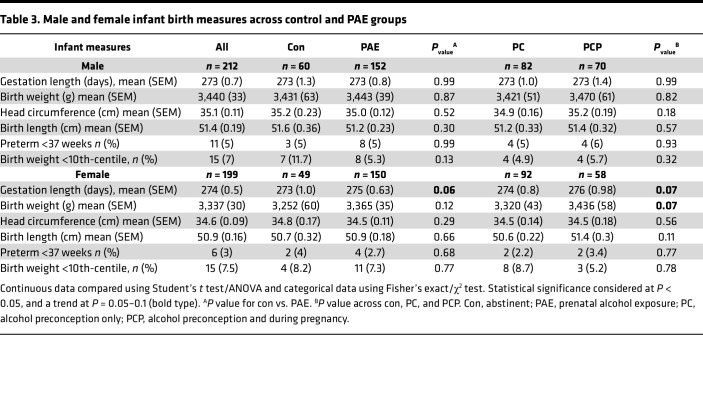
Male and female infant birth measures across control and PAE groups

**Table 4:**
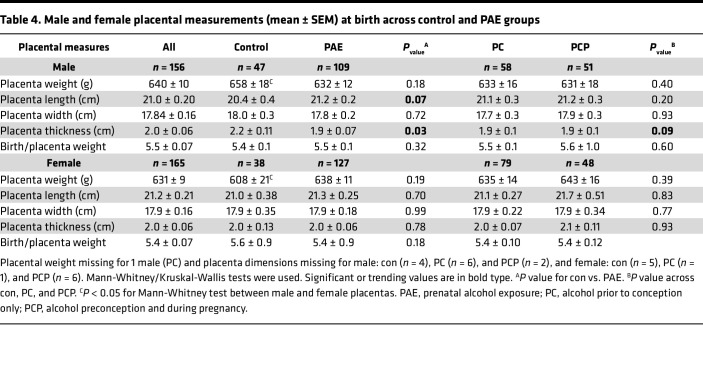
Male and female placental measurements (mean ± SEM) at birth across control and PAE groups

**Table 5:**
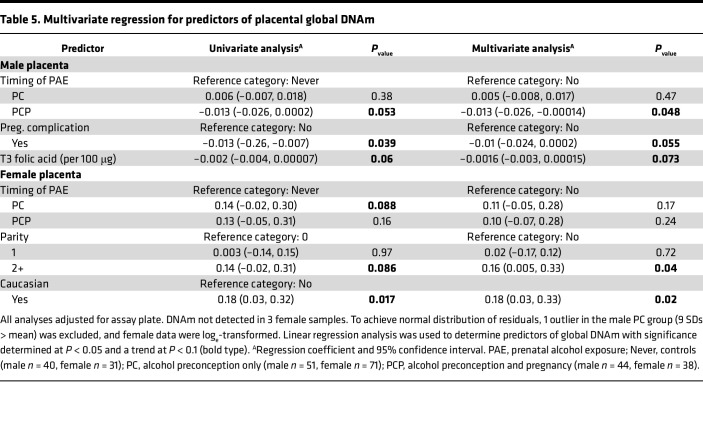
Multivariate regression for predictors of placental global DNAm

## Version 1 02/10/2025

Electronic publication
