# Supplementary material for: Prenatal alcohol exposure is associated with altered feto-placental blood flow and sex-specific placental changes
Source: JCI Insight. 2025 Feb 10;10(3):e186096. doi: 10.1172/jci.insight.186096 (PMC11948586; doi:10.1172/jci.insight.186096)
Supplement: Supplemental data [file jciinsight-10-186096-s194.pdf]

Supplemental Table 1 Alcohol consumption during pregnancy by period of exposure

PCP group (n = 128) by period of alcohol consumption					
**n**	**PC**	**T1**	**T2**	**T3**	**T1/T2 (AES only)**
65	✓	✓			
20	✓	✓	✓		
10	✓	✓	✓	✓	
4	✓	✓		✓	
7	✓		✓		
9	✓			✓	
2	✓		✓	✓	
3				✓	
1			✓	✓	
1		✓			
6					✓

PCP = alcohol prior to conception and during pregnancy, PC = prior to conception, T = trimester, AES = Australian eating survey completed at 24 weeks of gestation and included questions on alcohol consumption over the first 6 months of pregnancy.

Supplemental Table 2 Maternal intake of folate and choline from the diet

	**All**	**Con**	**PAE**	**p_{value}^{A}**	**PC**	**PCP**	**p_{value}^{B}**
**Diet survey n (%)[3]**	**346 (100)**	**84 (24.3)**	**262 (75.7)**		**154 (44.5)**	**108 (31.2)**	
folate µg /day mean (SEM)	483 (9.6)	452 (19.2)	493 (10.9)	**0.05**	501 (14.2)	481 (17.2)	0.10
< 600 µg /day, n (%)	275 (79.5)	70 (83)	205 (78)	0.35	119 (77)	86 (80)	0.54
≥ 600 µg /day, n (%)	71 (20.5)	14 (17)	57 (22)		35 (23)	22 (20)	
choline mg/day mean (SEM)	371 (6.7)	353 (14.5)	377 (7.5)	0.12	382 (9.9)	370 (11.5)	0.22
< 440 mg/day, n (%)	246 (71)	64 (77)	182 (70)	0.53	105 (68)	77 (71)	0.40
≥ 440 mg/day, n (%)	100 (29)	20 (23)	80 (30)		49 (32)	31 (29)	

Con=abstinent, PAE=prenatal alcohol exposure, PC=alcohol preconception only, PCP=alcohol preconception and during pregnancy. [A]P value for con vs PAE. [B]P value across con, PC and PCP. Continuous variables were compared using t-test/ANOVA for parametric data and Mann-Whitney/Kruskal-Wallis test for non-parametric data. Categorical data were compared using Fisher's exact/Chi-squared test. Statistical significance was determined at p<0.05 and a trend at p=0.05–0.1. Data on typical dietary consumption of 120 food and beverage items over the first 6 months of pregnancy was self-reported using the Australian Eating Survey and folate and choline intake estimated using the AUSNUT database with a choline content extension.

Supplemental Table 3 Maternal plasma concentrations of folate and choline at 28- and 36-weeks of gestation

Maternal plasma	All	Con	PAE	p_{value}^{A}	PC	PCP	p_{value}^{B}
**28 weeks gestation**	**225 (100)**	**59 (26.2)**	**166 (73.8)**		**90 (40)**	**76 (33.8)**	
Plasma folate categories, n (%):							
Possible Deficient <13.5nM	5 (2.2)	1 (1.7)	4 (2.4)	0.16[C]	1 (1.1)	3 (3.9)	0.34[C]
Normal 13.5–45.3nM	145 (64.5)	34 (57.6)	111 (66.9)		60 (66.7)	51 (67.1)	
Elevated >45.3nM	75 (33.3)	24 (40.7)	51 (30.7)		29 (32.2)	22 (29)	
folate (nM), mean (SEM)	40.6 (1.3)	41.0 (2.5)	40.4 (1.5)	0.68	40.4 (1.9)	40.5 (2.2)	0.92
choline (µM), mean (SEM)	10.7 (0.26)	10.8 (0.49)	10.6 (0.31)	0.80	10.8 (0.44)	10.4 (0.44)	0.91
**36 weeks gestation**	**178 (100)**	**45 (25.8)**	**133 (74.2)**		**73 (41)**	**60 (33.2)**	
Plasma folate categories, n (%):							
Possible Deficient <13.5nM	3 (1.7)	1 (2.2)	2 (1.5)	0.95[C]	2 (2.7)	0 (0)	0.99[C]
Normal 13.5–45.3nM	53 (29.8)	13 (28.9)	40 (30.1)		21 (28.8)	19 (31.7)	
Elevated >45.3nM	122 (68.5)	31 (68.9)	91 (68.4)		50 (68.5)	41 (68.3)	
folate (nM), mean (SEM)	63.4 (2.3)	64.3 (5.3)	63.1 (2.5)	0.78	62.9 (3.6)	63.3 (3.6)	0.99
choline (µM), mean (SEM)	10.1 (0.21)	9.7 (0.36)	10.3 (0.26)	0.34	10 (0.32)	10.6 (0.41)	0.36

Con=abstinent, PAE=prenatal alcohol exposure, PC=alcohol preconception only, PCP=alcohol preconception and during pregnancy. [A]p value for con vs PAE. [B]p value across con, PC and PCP. Continuous variables were compared using t-test or ANOVA for parametric data and Mann-Whitney U test or Kruskal-Wallis test for non-parametric data. Categorical data were compared using Fisher's exact test or Chi-squared test. Statistical significance was determined at $p<0.05$ and a trend at $p = 0.05–0.1$. [C]possible deficient groups had low numbers and were therefore combined with the normal groups for analysis using Chi-squared test. Plasma folate categories according to the World Health Organisation.

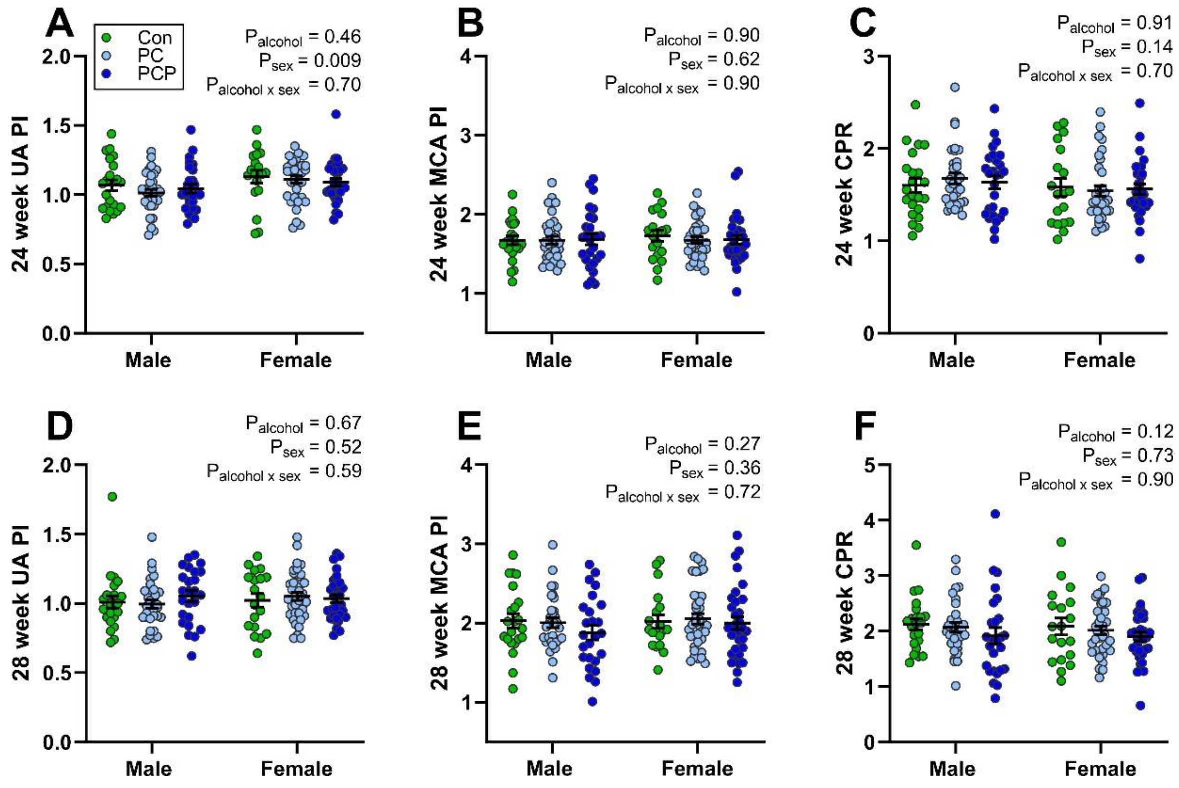

Supplemental Figure 1 Doppler ultrasound measures at three gestational time points in a general pregnancy cohort. Doppler ultrasound measurements of umbilical artery pulsatility index (UAPI), fetal middle cerebral artery pulsatility index, (MCAPI) and the ratio of MCAPI:UAPI (cerebroplacental ratio; CPR) at 24-weeks (**A–C**) and 28-weeks (**D–F**) of gestation in male and female fetuses from women with no alcohol consumption (Con, green), women reporting alcohol only prior to conception (PC, light blue) or alcohol prior to conception and during pregnancy (PCP, dark blue). Data are expressed as mean ± SEM. Measurements were compared using two-way ANOVA and Tukey's post hoc test, with statistical significance determined at $p<0.05$. Sample size (n) at 24 weeks in male: con (n=22), PC (n=32), PCP (n=27) and female: con (n=19), PC (n=38), PCP (n=32), at 28 weeks in male: con (n=22), PC (n=32), PCP (n=25) and female: con (n=19), PC (3 n=4), PCP (n=32).

Supplemental Table 4 Univariate regression analysis of potential predictors of cerebroplacental ratio

	Cerebroplacental Ratio	
**Predictor**	**Unadjusted estimate (95% CI)**	$\mathbf{p_{value}}$
PAE	*Reference category: No/control*	
Yes	-0.15 (-0.33, 0.039)	0.12
Period of alcohol exposure	*Reference category:*	
PC	-0.06 (-0.26, 0.14)	0.56
PCP	-0.25 (-0.46, -0.04)	**0.02**
Caucasian	*Reference category: No*	
Yes	0.08 (-0.10, 0.26)	0.41
Smoker	*Reference category: No*	
Yes	0.03 (-0.31, 0.36)	0.88
Spontaneous conception	*Reference category: No*	
Yes	0.05 (-0.28, 0.37)	0.78
Parity	*Reference category: 0*	
1	0.14 (-0.04, 0.32)	0.12
2+	0.23 (-0.008, 0.47)	**0.06**
Planned pregnancy	*Reference category: No*	
Yes	-0.02 (-0.22, 0.18)	0.83
Pregnancy complications[A]	*Reference category: No*	
Yes	0.03 (-0.19, 0.25)	0.78
Spontaneous onset of labour	*Reference category: No*	
Yes	-0.08 (-0.24, 0.09)	0.35
Preconception folic acid supplement	*Reference category: No*	
Yes	0.12 (-0.05, 0.29)	0.16
T1 folic acid supplement	*Reference category: No*	
Yes	0.21 (-0.08, 0.50)	0.15
T2 folic acid supplement	*Reference category: No*	
Yes	0.09 (-0.13, 0.31)	0.43
T3 folic acid supplement	*Reference category: No*	
Yes	0.1 (-0.12, 0.32)	0.39
Dietary folate (µg/day)	0.0002 (-0.0003, 0.0008)	0.41
Dietary choline (mg/day)	0.0000 (-0.0007, 0.0008)	0.94
Maternal plasma folate 28w (nM)	-0.003 (-0.007, 0.001)	0.19
Maternal plasma folate 36w (nM)	-0.0003 (-0.003, 0.002)	0.82
Maternal plasma choline 28w (µM)	0.018 (-0.005, 0.04)	0.13
Maternal plasma choline 36w (µM)	0.003 (-0.27, 0.32)	0.86
Pre-pregnancy BMI (kg/m^2)	-0.001 (-0.017, 0.015)	0.88
Age at conception (years)	0.008 (-0.009, 0.024)	0.38
Gestation length (days)	0.004 (-0.006, 0.015)	0.42

PAE=Prenatal alcohol exposure, PC=alcohol preconception only, PCP=alcohol preconception and pregnancy

[A]gestational diabetes, hypertension, pre-eclampsia, cholestasis, intra uterine growth restriction, preterm prelabour rupture of membranes, antepartum haemorrhage. Residuals visually inspected for normal distribution using diagnostic distributional plots.

Supplemental Table 5 Maternal characteristics in women with CPR above and below the 5th centile

Maternal characteristics	<5th centile (n = 24)	>5th centile (n = 151)	p_{value}
PAE n (%)			
PC	11 (45.8)	60 (39.7)	**0.01**
PCP	12 (50)	47 (31.1)	
No	1 (4.2)	44 (29.1)	
Caucasian n (%)			
Yes	19 (79.2)	1050(69.5)	0.47
No	5 (20.8)	46 (30.5)	
Smoker[A] n (%)		[B]	
Yes	2 (8.3)	9 (6)	0.56
No	22 (91.7)	141 (94)	
Spontaneous conception n (%)		[C]	
Yes	20 (83.3)	139 (94.6)	**0.07**
No	4 (16.7)	8 (5.4)	
Parity n (%)			
0	15 (62.5)	64 (42.4)	**0.08**
1+	9 (37.5)	87 (57.6)	
Planned pregnancy n (%)		[C]	
Yes	18 (75)	115 (80)	0.79
No	6 (25)	32 (20)	
Pregnancy complications [#] n (%)			
Yes	3 (12.5)	27 (17.9)	0.77
No	21 (87.5)	124 (82.1)	
Spontaneous onset of labour n (%)		[B]	
Yes	15 (62.5)	72 (47.7)	0.27
No	9 (37.5)	79 (52.3)	
Pre-pregnancy BMI (kg/m^2) mean (SD)	24 (5.3)	24 (5.1)	0.75
Age at conception (years) mean (SD)	32 (4.6)	31.5 (4.9)	0.49
Gestation length (days) mean (SD)	273 (8.6)	275 (7.7)	0.22
Preconception folic acid supplement n (%)			
No	13 (54)	51 (34)	**0.05**
Yes	11 (46)	100 (66)	
T1 folic acid supplement n (%)			
No	3 (12.5)	12 (8)	0.46
Yes	21 (87.5)	139 (92)	
T2 folic acid supplement n (%)			
No	5 (21)	23 (15)	0.487
Yes	19 (79)	128 (85)	
T3 folic acid supplement n (%)			
No	4 (17)	25 (17)	0.99
Yes	20 (83)	126 (83)	
**Dietary survey completed**	**n = 19**	**n = 107**	
Dietary folate (µg/day), mean (SD)	503.1 (209.5)	483.2 (175.4)	0.71
Dietary choline (mg/day), mean (SD)	371.4 (135.2)	369.5 (128.0)	0.95
**Plasma samples 28-weeks gestation**	**n = 24**	**n = 147**	
Maternal plasma folate (nM), mean (SD)	46.9 (24.5)	40.4 (18.9)	0.38
Maternal plasma choline (µM), mean (SD)	11.6 (4.2) [B]	10.9 (3.6) [D]	0.56
**Plasma samples 36-weeks gestation**	**n = 24**	**n = 141**	
Maternal plasma folate (nM), mean (SD)	66.1 (34.2) [B]	65.0 (29.3)	0.97
Maternal plasma choline (µM), mean (SD)	10.1 (1.9) [B]	10.0 (3.0)	0.34

CPR=cereboplacental ratio; PAE = prenatal alcohol exposure; PC = preconception only, PCP = prior to conception and during pregnancy. T=trimester. [A]smoked in the 12 weeks preconception or during pregnancy, [B]1 missing, [D]4 missing [C]3 missing. [E]gestational diabetes, gestational hypertension, pre-eclampsia, cholestasis, intra uterine growth restriction, preterm prelabour rupture of membranes, antepartum haemorrhage. Continuous variables were compared using unpaired t-test/one-way ANOVA where residuals were normally distributed or where transformation of data allowed normal distribution of residuals. Otherwise, Mann-Whitney/Kruskal-Wallis tests were used. Categorical data were compared using Fisher's exact test or Chi-squared test.

Supplemental Table 6 Logistic regression for predictors of cerebroplacental ratio <5th centile

	Odds of CPR <5th centile	
**Predictor**	**Unadjusted OR (95% CI)**	**p_{value}**
Period of PAE	*Reference category: Control*	
PC	8.1 (1.0, 64.8)	0.050
PCP	11.2 (1.4, 90.0)	0.023
Parity	*Reference category: 1+*	
0	2.3 (0.93, 5.50)	0.071
Preconception folic acid	*Reference category: Yes*	
No	2.3 (0.97, 5.54)	0.059
Spontaneous conception	*Reference category: Yes*	
No	3.5 (0.96, 12.61)	0.058

PAE = prenatal alcohol exposure; PC = preconception only, PCP = preconception and during pregnancy

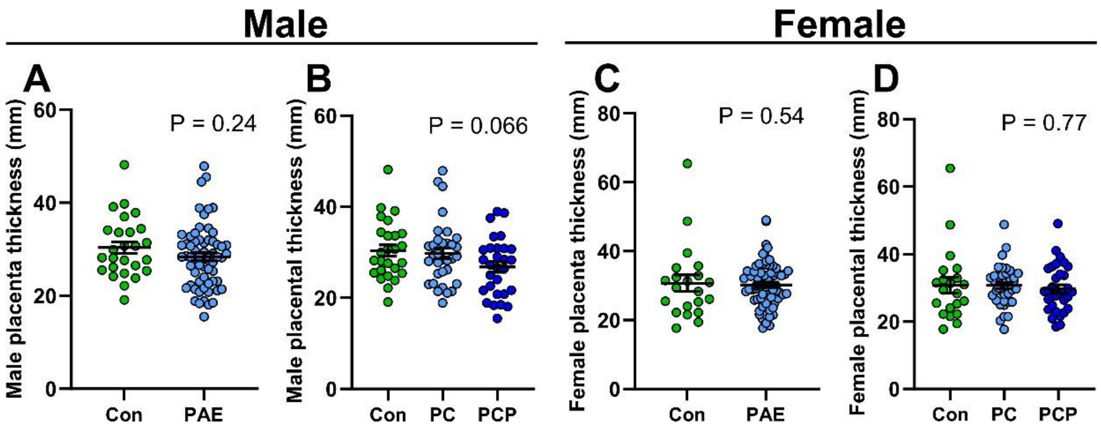

Supplemental Figure 2 Placental thickness at 36-weeks of gestation measured using ultrasound images. Mean placental thickness was compared across control and alcohol groups in male (**A** and **B**), and female placentas (**C** and **D**). Doppler ultrasound images were collected from women with no prenatal alcohol (Con: male n=26, female n=21), or with prenatal alcohol exposure (PAE: male n=62, female n=70). The PAE group was also divided into women who reported alcohol only preconception (PC: male n=33, female n=38) or alcohol preconception and during pregnancy (PCP: male n=29, female n=32). Data are expressed as mean ± SEM. Control and PAE groups were compared using unpaired t-test/one-way ANOVA where residuals were normally distributed or where transformation of data allowed normal distribution of residuals. Otherwise, Mann-Whitney/Kruskal-Wallis tests were used. $p<0.05$ was considered statistically significant and p=0.05–0.1 considered a trend.

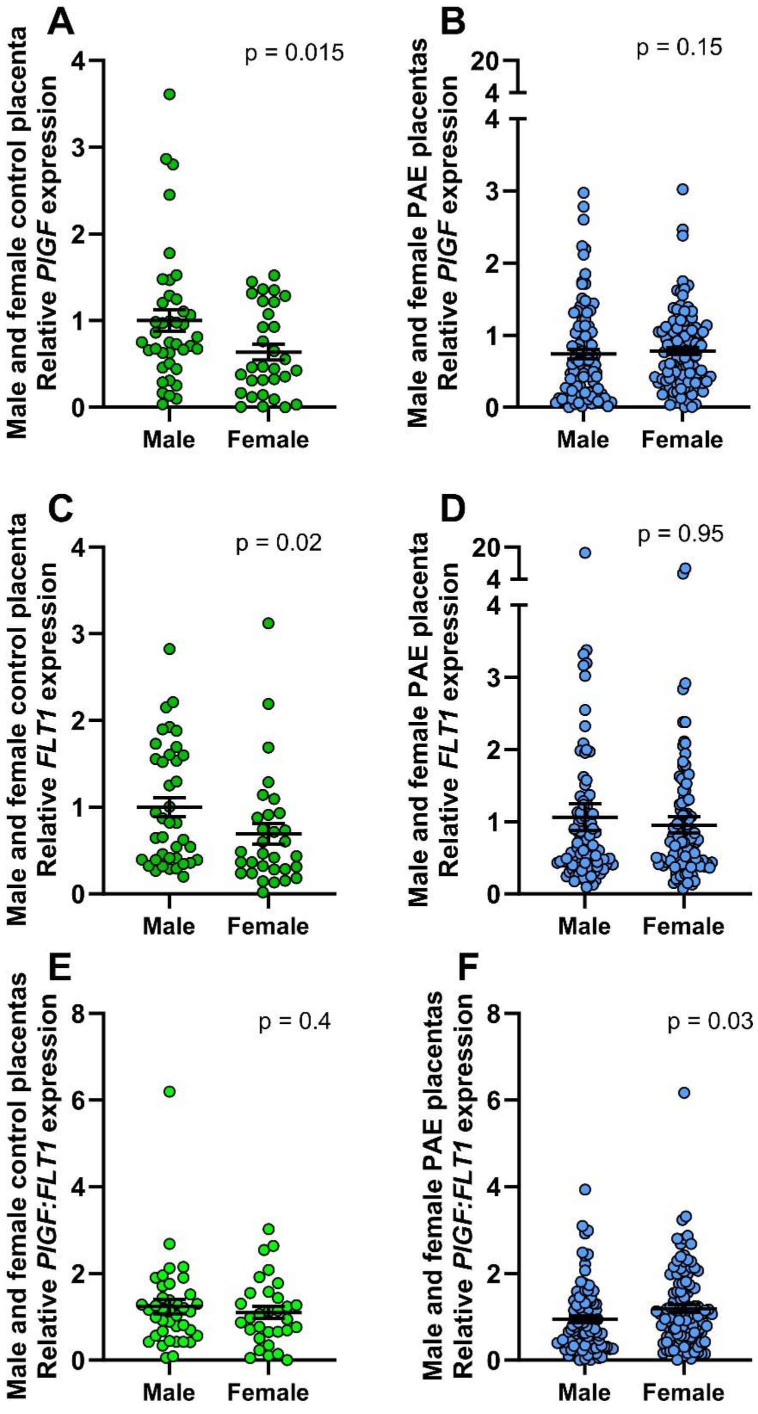

Supplemental Figure 3 Sexually dimorphic placental expression of growth factors. Mean expression of PlGF (**A** and **B**), FLT1 (**C** and D) and the ratio of PlGF:FLT1 (**E** and **F**) in male and female placentas from control/abstinent women and women with PAE. Placentas were collected from women who reported no prenatal alcohol (Con: male n=40, female con n=31) or prenatal alcohol exposure (PAE: male n=95, female n=112). Gene expression was normalised to *ACTB* endogenous control, and fold-change expressed relative to the male Con group. Data are expressed as mean ± SEM. Male and female data were compared using unpaired t-test/one-way ANOVA where residuals were normally distributed or where transformation of data allowed normal distribution of residuals. Otherwise, Mann-Whitney/Kruskal-Wallis tests were used. $p<0.05$ was considered statistically significant and p=0.05–0.1 considered a trend.

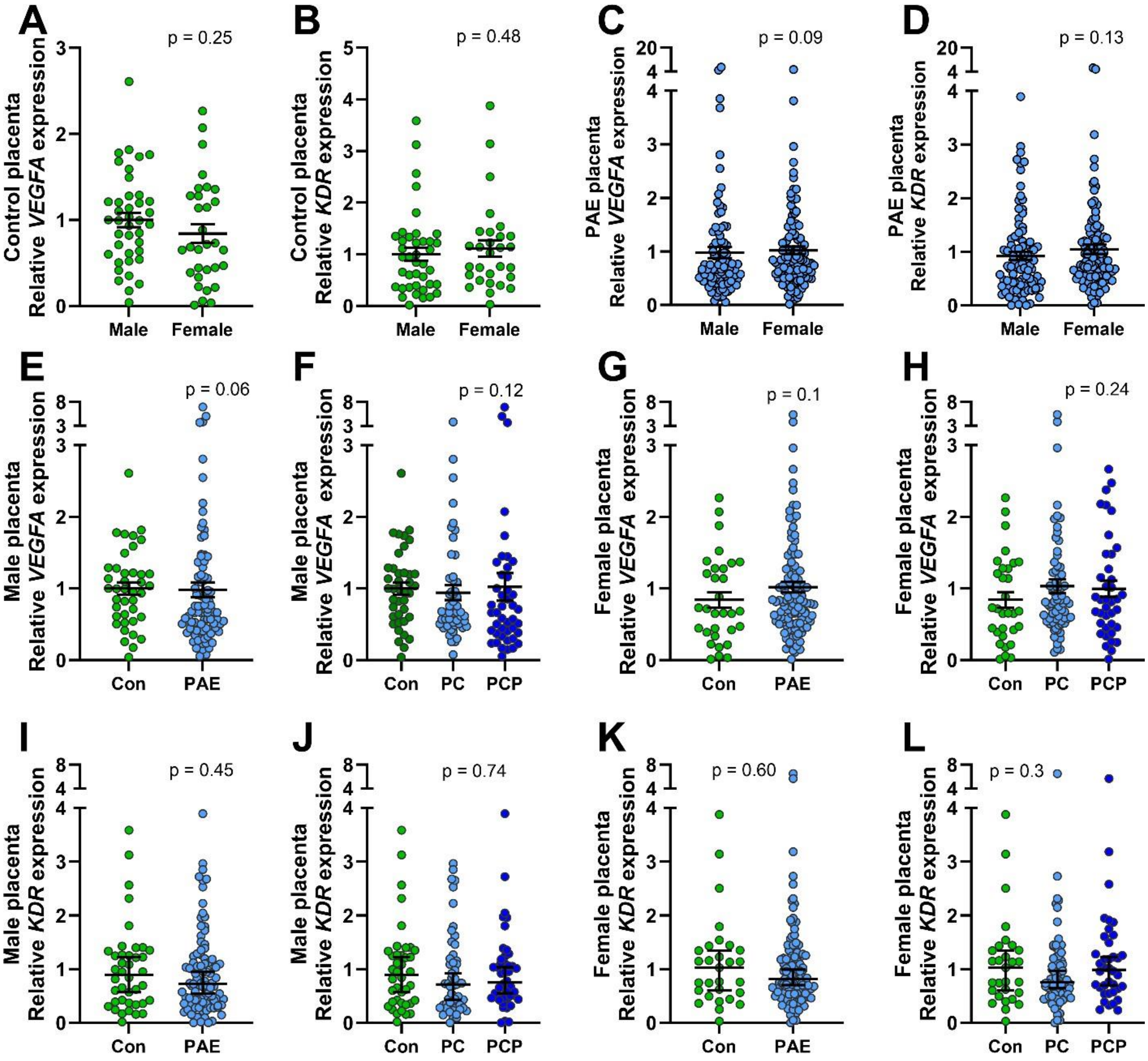

Supplemental Figure 4 Expression of placental growth factors. mRNA expression of vascular endothelial growth factor A (*VEGFA*) and kinase insert domain receptor (*KDR*) in male and female placentas from control/abstinent women (**A and B**), and women with PAE (**C** and **D**), and across control and PAE groups in male (E, F, I, J) and female (**G**, **H**, **K** and **L**) placentas. Placentas were collected from women who reported no prenatal alcohol (Con: male n=40, female con n=31) or prenatal alcohol exposure (PAE: male n=95, female n=112). The PAE group was also divided into women who reported alcohol only prior to conception (PC: male n=51, female n=73) or alcohol prior to conception and during pregnancy (PCP: male n=44, female n = 39). Gene expression was normalised to *ACTB* endogenous control, and fold-change expressed relative to the male Con group. Data are expressed as mean ± SEM. Control and PAE groups were compared using unpaired t-test/one-way ANOVA where residuals were normally distributed or where transformation of data allowed normal distribution of residuals. Otherwise, Mann-Whitney/Kruskal-Wallis tests were used. $p<0.05$ was considered statistically significant and p=0.05–0.1 considered a trend. Below detection limit: VEGFA for male con (n=1) and female PC (n=1), PCP (n=1); KDR for female con (n=2), PC (n=2), PCP (n=2).

Supplemental Table 7 Linear regression for predictors of placental global DNAm

Maternal characteristics	Male %5mc	p_{value}	Female ($\log_e$)%5mc	p_{value}
	**Plate adjusted estimate**[A]		**Plate adjusted estimate**[A]	
PAE	*Reference category: No*		*Reference category: No*	
Yes	-0.003 (-0.15, 0.01)	0.61	0.14 (-0.16, 0.29)	**0.078**
Period of alcohol exposure	*Reference category: Never*		*Reference category: Never*	
PC	0.006 (-0.007, 0.02)	0.38	0.14 (-0.02, 0.30)	**0.088**
PCP	-0.13 (-0.26, 0.0002)	**0.053**	0.13 (-0.05, 0.31)	0.16
Caucasian	*Reference category: No*		*Reference category: No*	
Yes	0.0007 (-0.011, 0.013)	0.90	0.18 (0.03, 0.32)	**0.02**
Smoker*	*Reference category: No*		*Reference category: No*	
Yes	-0.013 (-0.04, 0.02)	0.43	0.17 (-0.08, 0.41)	0.19
Spontaneous conception	*Reference category: No*		*Reference category: No*	
Yes	0.004 (-0.015, 0.02)	0.70	-0.015 (-0.24, 0.21)	0.89
Parity	*Reference category: 0*		*Reference category: 0*	
1	0.004 (-0.007, 0.016)	0.49	0.003 (-0.14, 0.15)	0.97
2+	-0.0001 (-0.015, 0.015)	0.99	0.14 (-0.02, 0.31)	**0.086**
Planned pregnancy	*Reference category: No*		*Reference category: No*	
Yes	-0.003 (-0.17, 0.01)	0.63	-0.1 (-0.25, 0.05)	0.18
Pregnancy complications[B]	*Reference category: No*		*Reference category: No*	
Yes	-0.13 (-0.26, -0.0007)	**0.04**	-0.07 (-0.24, 0.10)	0.40
Spontaneous labour	*Reference category: No*		*Reference category: No*	
Yes	0.0047 (-0.006, 0.015)	0.39	-0.03 (-0.16, 0.11)	0.71
Pre-pregnancy BMI (Kg/m^2)	-0.0007 (-0.002, 0.003)	0.19	0.007 (-0.005, 0.019)	0.26
Age at conception (yrs)	-0.0002 (-0.0015, 0.001)	0.70	0.001 (0.015, 0.013)	0.84
Gestation length (days)	-0.0001 (-0.0007, 0.0005)	0.80	-0.006 (-0.15, 0.002)	0.16
PC folic acid (per 100µg)	-0.0006 (-0.002, 0.0004)	0.26	-0.0048 (-0.017, 0.007)	0.42
T1 folic acid (per 100µg)	6.8 e^{-6} (-0.001, 0.001)	0.99	-0.0028 (-0.012, 0.006)	0.54
T2 folic acid (per 100µg)	-0.0004 (-0.001, 0.0006)	0.45	-0.0048 (-0.015, 0.005)	0.36
T3 folic acid (per 100µg)	-0.002 (-0.004, 0.00007)	**0.06**	-0.0066 (-0.019, 0.007)	0.32
Dietary folate (µg/day)	9.97 e^{-6} (-24.4 e^{-6}, 44.4 e^{-6})	0.57	34 e^{-6} (-392 e^{-6}, 460 e^{-6})	0.88
Dietary choline (mg/day)	38.2 e^{-6} (-13.5 e^{-6}, 89.8 e^{-6})	0.15	256 e^{-6} (-327 e^{-6}, 840 e^{-6})	0.39
Plasma folate 28w (nM)	-47.8 e^{-6} (-346 e^{-6}, 250 e^{-6})	0.75	0.001 (-0.003, 0.005)	0.56
Plasma choline 28w (µM)	-0.0002 (-0.002, 0.001)	0.76	-0.004 (-0.03, 0.02)	0.72
Plasma plasma folate 36w (nM)	79.6 e^{-6} (-125 e^{-6}, 285 e^{-6})	0.44	-0.0002 (-0.003, 0.003	0.92
Plasma choline 36w (µM)	0.0002 (-0.003, 0.003)	0.87	-0.008 (-0.04, 0.21)	0.60

PAE=Prenatal alcohol exposure, PC=preconception only, PCP=preconception and during pregnancy, BMI=body mass index. T=trimester. [A]Assay plate number was included as a covariate in all analyses. *Smoked preconception and/or during pregnancy. [B]gestational diabetes, gestational hypertension, pre-eclampsia, cholestasis, intra uterine growth restriction, preterm prelabour rupture of membranes, antepartum haemorrhage.

Table 8 Primers used for qPCR

Gene symbol	Gene name	Function in the placenta	Primer sequence 5' – 3'
PlGF	Placental growth factor	Growth	F- AGCTCCTAAAGATCCGTTC<br>R- GACGGTAATAAATACACGAGC
VEGFA	Vascular endothelial growth factor A	Growth	F- AATGTGAATGCAGACCAAAG<br>R- GACTTATACCGGGATTTCTTG
VEGFR1	Vascular endothelial growth factor Receptor 1 (*FLT1*)	Growth	F- ATGTGAAACCCCAGATTTAC<br>R- TGATTGTAGGTTGAGGGATAC
VEGFR2	VEGF Receptor 2 (*KDR*)	Growth	F- GTACATAGTTGTCGTTGTAGG<br>R- TCAATCCCCACATTTAGTTC
DNMT1	DNA methyltransferase 1	Maintenance DNAm	F- CGTAAAGAAGAATTATCCGAGG<br>R- GTTTTCTAGACGTCCATTCAC
DNMT3A	DNA methyltransferase 3A	De novo DNAm	F- ATTACTACGAGGTCAAACTCC<br>R- GGGAAACCAAATACCCTTTC
DNMT3B	DNA methyltransferase 3B	De novo DNAm	F- CTTACCTTACCATCGACCTC<br>R- ATCCTGATACTCTGAACTGTC
SLC19A1	Reduced Folate Carrier 1 (*RFC*)	Folate transport	F- GTCAAGACCATCATCACTTTC<br>R- ACATGCGTTCTTCATTCTG
mTOR	Mechanistic target of rapamycin	Nutrient sensor	F- GGAGGAGAAATTTGATCAGG<br>R- GGGCAACAAATTAAGGATTG

Supplemental methods

Folic acid intake from supplements

Where the frequency options for prenatal supplements 'never', 'varies/as required' or '<once/week' were selected, a value of zero was assigned. '1 to 2 times/week' was assigned as 2 times per week and '3 or more times/week' was assigned 3 times/week. Where a supplement brand could not be recalled, a conservative estimate was applied using the minimum folic acid content of all brands reported by participants in this study, which was 400µg.

Calculation of dietary intake of choline and folate

For each food and beverage item selected in the Australian Eating Survey, frequency options of 'never' or '< once per month' were assigned zero. Where frequency options provided a range e.g '2-4 times per month', an average of 3 times/month was assigned and where an option such as '4 or more times per week' was selected, a value of 4 times per week was assigned. Intake was calculated over a month and divided by 31 to obtain mean intake/day. Total folate intake from both the diet (including fortification) and folic acid supplements was calculated as total DFE = µg dietary folate + (1.7 x µg folic acid). This accounts for the higher bioavailability of folic acid (85%) compared with naturally occurring food folate (50%).

Plasma choline measurement

To generate a standard curve, a pooled plasma sample was dialysed (Slide- a-Lyzer cassette, 10KDa cut off, Thermo Scientific #66380) against phosphate buffered saline (pH 7.4) containing 4mM EDTA overnight at 4^0C, to remove endogenous choline. Aliquots of dialysed plasma were spiked with choline to a final concentration of 25, 12.5, 6.25, 3.125, 1.56 and 0 µM. Samples and standards were precipitated with 3 volumes of ice cold 100% acetonitrile containing d9-choline as the internal standard (10µM final), vortexed, incubated on ice for 10 mins and centrifuged at 4^0C, 14,000 x *g* for 10 min. The supernatant was removed, and centrifugation repeated before transferring the clear supernatant into a vial for analysis. Chromatographic separation was achieved using a C18 column under isocratic conditions using mobile phase A (1% acetonitrile, 0.1% formic acid) and mobile phase B (90% acetonitrile, 0.1% formic acid) with a flow rate of 400 µL/min. The standard curve was linear over the 0 - 25 µM range (r^2 = 0.996). Eight samples were precipitated and analysed in triplicate to calculate the coefficient of variation (CV), which was 16.0%.

Plasma folate measurement

Any samples above the assay range (1.36 – 45.4nM) were diluted 1:1 with Diluent Universal (cat# 5192943190), and further dilution up to a maximum of 1:4 where necessary. Twenty samples were measured in duplicate to determine the CV (4.7%).

Gene expression

RNA concentration and purity were measured using a Nanodrop2000 spectrophotometer (ThermoFisher Scientific). The 260:280 ratio for all samples was >2.0. RNA integrity was assessed using the RNA 6000 Nano Kit (Cat# 5067-1511, Integrated sciences, NSW,

Australia) and an Agilent 2100 bioanalyser (Agilent Technologies, Vic, Australia) for a random subset of 8 samples (RIN>5.8).

Global DNAm

A standard curve (0% – 2% 5mC) was generated by diluting positive control DNA (10% 5mC, 50ng/µl) with negative control DNA (0% 5mC, 50ng/µl). 100 ng of methylated standards or gDNA samples were added to a 96-well plate containing binding buffer. The methylated portion of DNA was detected using an anti-5-methylcytosine (5mC) capture antibody and a detection antibody allowing colorimetric quantification. Control and PAE samples were distributed evenly across 4 plates. Each plate contained a standard curve in duplicate and the same four samples assayed in triplicate allowing calculation of the intra-assay CV (8.1%) and inter-assay CV (15.4%).
